# The Impact of Different Levels of Adaptive Iterative Dose Reduction 3D on Image Quality of 320-Row Coronary CT Angiography: A Clinical Trial

**DOI:** 10.1371/journal.pone.0125943

**Published:** 2015-05-06

**Authors:** Sarah Feger, Matthias Rief, Elke Zimmermann, Peter Martus, Joanne Désirée Schuijf, Jörg Blobel, Felicitas Richter, Marc Dewey

**Affiliations:** 1 Department of Radiology, Charité Medical School, Humboldt-Universität zu Berlin, Freie Universität Berlin, Berlin, Germany; 2 Department of Radiology, Charité Medical School, Berlin, Germany; 3 Toshiba Medical Systems Europe B.V., Zoetermeer, the Netherlands; 4 Department of Medical Statistics, Informatics and Documentation, University Hospital of Friedrich-Schiller University Jena, Jena, Germany; Sapienza University of Rome, ITALY

## Abstract

**Purpose:**

The aim of this study was the systematic image quality evaluation of coronary CT angiography (CTA), reconstructed with the 3 different levels of adaptive iterative dose reduction (AIDR 3D) and compared to filtered back projection (FBP) with quantum denoising software (QDS).

**Methods:**

Standard-dose CTA raw data of 30 patients with mean radiation dose of 3.2 ± 2.6 mSv were reconstructed using AIDR 3D mild, standard, strong and compared to FBP/QDS. Objective image quality comparison (signal, noise, signal-to-noise ratio (SNR), contrast-to-noise ratio (CNR), contour sharpness) was performed using 21 measurement points per patient, including measurements in each coronary artery from proximal to distal.

**Results:**

Objective image quality parameters improved with increasing levels of AIDR 3D. Noise was lowest in AIDR 3D strong (p≤0.001 at 20/21 measurement points; compared with FBP/QDS). Signal and contour sharpness analysis showed no significant difference between the reconstruction algorithms for most measurement points. Best coronary SNR and CNR were achieved with AIDR 3D strong. No loss of SNR or CNR in distal segments was seen with AIDR 3D as compared to FBP.

**Conclusions:**

On standard-dose coronary CTA images, AIDR 3D strong showed higher objective image quality than FBP/QDS without reducing contour sharpness.

**Trial Registration:**

Clinicaltrials.gov NCT00967876

## Introduction

320-detector row coronary computed tomography angiography (CTA) shows a high diagnostic accuracy in the diagnosis of coronary artery disease (CAD) with reduced effective dose as compared to conventional coronary angiography, and it is characterised by a high spatial and temporal resolution [1, 2]. Optimal image quality is necessary to reliably evaluate the coronary arteries. Accordingly, a compromise should be realised between radiation exposure and image quality that allows the thorough analysis of the coronary arteries of patients with suspected CAD. The reduction of radiation dose is associated with an increase of noise and, as a result, a deterioration of image quality [3]. Currently, the radiation exposure of a CTA performed with 320-row CT ranges between 2–5 mSv [1, 4] and routinely, filtered back projection (FBP) is used to reconstruct the dataset. To improve the trade-off between image quality and radiation dose, noise reduction filters such as quantum denoising software (QDS) can be applied in conjunction with FBP reconstruction [5].

More recently, various iterative reconstruction algorithms have been introduced [6–8]. Some of the available iterative reconstruction algorithms mainly work in the image data domain, such as the adaptive statistical iterative reconstruction (ASIR) and the iterative reconstruction in image space (IRIS) [9, 10]. Besides, there are also iterative reconstructions that predominantly work in the raw data domain, such as the sinogram-affirmed iterative reconstruction (SAFIRE) and hybrid iterative reconstruction (HIR) [11–13]. AIDR 3D is designed to work in both the raw data and the image data domain [14–16]. There are two parts in the process. First, the algorithm uses scanner specific parameters (reconstruction filter, slice thickness etc.) and a statistical noise model together with projection noise estimation in the raw data domain to reduce photon and electric noise. Second, the initially calculated reference image is inserted into the model-based iteration cycle, taking the body region and kernel into consideration. After each iterative cycle, the output image is being compared to the reference image and blending of both parts in the process is applied. Three different user pre-set levels of AIDR 3D are available from mild over standard to strong, increasing the strength of the noise reduction processing in the raw data domain as well as the number of iterations in the image data domain.

Recent studies have compared the effects of different levels of iterative reconstructions on image quality to conventional FBP [17–21]. Most of these studies show a reduction of noise, while contrast-to-noise ratio (CNR) is improved by increasing strength of the noise filter. Currently, and to our knowledge, data comparing image quality between the different levels of AIDR 3D is limited. Moreover, the potential benefit of applying the different levels of AIDR 3D instead of the previously existing option FBP in combination with QDS reconstruction in standard dose coronary CTA acquisitions remains to be determined.

The aim of this study is the systematic image quality evaluation of three different available AIDR 3D levels (mild, standard and strong) as compared to the combination of FBP and QDS for standard dose coronary CTA by using different quantitative parameters: signal intensity/attenuation (CT number [HU]), noise, signal-to-noise ratio (SNR), CNR and contour sharpness.

## Materials and Methods

### Ethics statement

This clinical trial has been registered (***www.clinicaltrials.gov***; Study NCT00967876; August 27, 2009). The study is based on the Declaration of Helsinki and in accordance with the TREND guidelines [22] because of the non-randomized study design. Find attached the TREND checklist (S1 Checklist). We received written informant consent from all patients for the study. The study protocol is available as S1 Protocol. It was approved by the Charité IRB (EA1/133/08) and the Federal Office for Radiation Protection (BfS Z5-22462/2-2008-057) and all authors confirm that all related trials for this intervention are registered.

### Study design and patient collective

**Fig 1:**
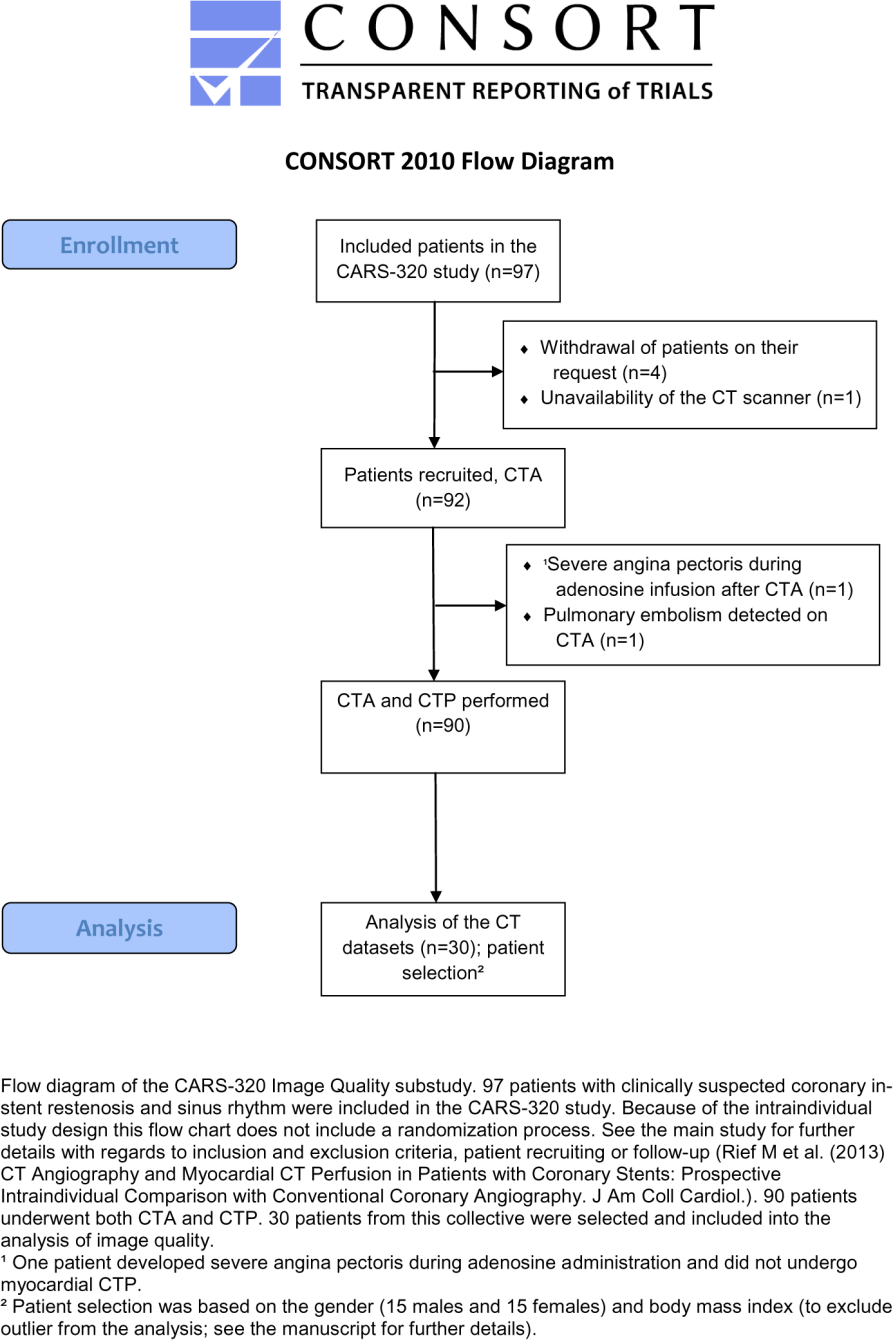
CONSORT Flow Diagram.

This study was a non-prespecified substudy of the prospective intention to diagnose study Coronary Artery Stent Evaluation with 320-row CT [23]. In this image quality substudy we included patients with suspected in-stent restenosis and stable symptomatic. **Fig 1**shows the CONSORT Flow Diagram of this study. Patient enrolment was between April 2, 2009 and November 23, 2011 (the slight delay in trial registration is due to administrative reasons; see www.clinicaltrials.gov for further details). Clinical follow-up was performed 6 months, 12 months and 24 months after CT examination (last follow-up in 11/2013). The included patients were selected with regards to their gender (15 male patients, 15 female patients) and their body mass index (BMI male 29.1±2.5 kg/m²; BMI female 28.5±5.2 kg/m²) that should exclude outliers from the analysis. All patients underwent a coronary CTA. The exclusion and inclusion criteria, the CT protocol and the clinical indications for the examinations have already been published [23, 24]. The study protocol was approved by the local ethics committees and all patients gave written informed consent after verbal and written information of the study.

### Coronary CT angiography (CTA)

CTA was performed on a 320-row CT (0.5 mm detector collimation and 350 ms rotation time, Aquilion ONE, Toshiba Medical Systems, Otawara Japan) and occurred within 14 days after the inclusion in the study. The CTA was performed with electrocardiogram (ECG) triggering covering 70–80% of the RR interval if the heart rate (HR) was ≤65 bpm, and in case of a HR ranging from 66 to 79 bpm ECG triggering was performed between 40–80% of the RR interval (no patient had a HR >79 bpm). Immediately before the scan, nitroglycerine was sublingually administered (1.2 mg Nitrolingual N Spray, Pohl-Boskamp, Hohenlockstedt, Germany). In case of a HR ≥65 bpm the patients received oral beta blockers one hour before CTA (atenolol, Tenormin, Astra-Zeneca), or directly before the scan beta blockers were intravenously administered (esmolol, Brevibloc, Baxter). The amount of the contrast agent (Iomeron 400, Bracco Imaging, Milan, Italy) was 50–70 ml, and the amount and the flow were adjusted to the patients`body weight [23, 24]. By using a tube voltage of 120 kV for each patient the remaining scan parameters were adapted to the patients`BMI [23]. The tube current in the study protocol varied between 250 mA and 450 mA.

### Reconstruction

The reconstruction was performed with a field of view (FOV) of 180 mm and a kernel of FC 05 for both IR 3D and FBP. Slice thickness was 0.5 mm by using a reconstruction interval of 0.25 mm. First, FBP/QDS was reconstructed using automated „BestPhase”reconstruction (PhaseXact, Toshiba, Tokyo, Japan) that automatically selects the phase with fewest motion influence in the 3D dataset [24, 25]. Additional reconstructions were conducted in 5% steps of the available RR interval. Subsequently, the phase with the least motion artefacts for the coronary arteries was visually chosen and reconstructed with IR 3D mild, IR 3D standard and IR 3D strong by using the reconstruction parameters as described above.

### Measurement points

**Fig 2:**
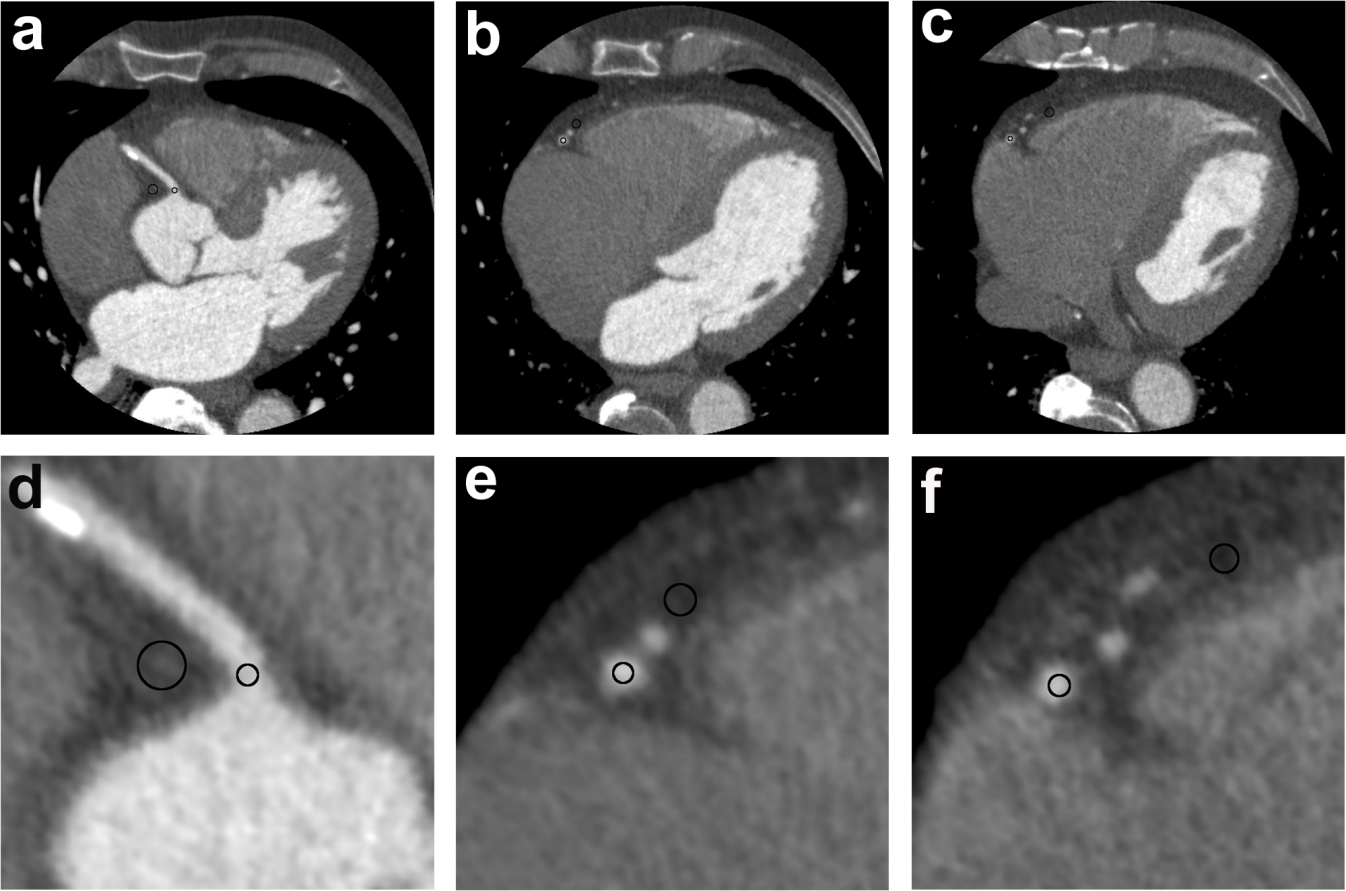
Analysis of signal and noise. Measurement in the vessel and the surrounding tissue at the example of right coronary artery (RCA) being reconstructed by using filtered back projection/ quantum denoising filtering software; axial slices; **a/d**: proximal RCA measurement point 5 mm distal of the beginning of the vessel; **b/e**: medial RCA measurement point 5 mm distal of the first branch; **c/f**: distal RCA measurement point 5 mm distal of the second branch; **a-c:** field of view of 180 mm; **d-f:** zooming of **a-c;** The ROIs in the vessel lumen were placed as large as possible without integrating calcified and non-calcified plaques, stents and also the vessel wall into the analysis (approximately 50% of the vessel lumen).

The data analysis was performed using a dedicated workstation (Vitrea fx, Toshiba, Tokyo, Japan). A total of 21 measurement points per patient was defined for the analysis. The first measurement point was placed in the ascending aorta directly cranial of the origin of the right coronary artery (RCA). Two additional measurement points were localised in the proximal left main coronary artery 5 mm behind the origin of the sinus of Valsalva in the vessel lumen and in the surrounding tissue at the same slice position as used for the measurement in the vessel (**Fig 2**). Per coronary artery (RCA, left anterior descending coronary artery [LAD], and left circumflex artery [LCX]) 6 measurement points were placed. The proximal measurement point was manually placed 5 mm distal of the origin of the vessel, the medial measurement point was placed 5 mm distal of the first branch in the major vessel, and the distal measurement point was located 5 mm distal of the second branch in the major vessel. The corresponding measurements in the surrounding tissue were performed at the same slice position and in spatial proximity to the measurement point in the vessel.

### Analysis of signal and noise

The analysis of signal and noise were performed by using a region of interest (ROI) placed into the vessel lumen and the corresponding surrounding tissue on axial images (**Fig 2**) for the 21 measurement points. The ROI for the measurement in the ascending aorta was standardised to an area of 1 cm². The signal intensity corresponded to the grey value. The noise was defined as the standard deviation of the signal intensity of the ROI in the vessel lumen or the epicardial tissue measurement point. The SNR was calculated as follows: signal intensity in the measurement point in HU divided by the noise in the ascending aorta. The CNR was calculated as the difference between the signal intensity in the epicardial tissue and the signal intensity in the corresponding vessel lumen in HU divided by the noise in the ascending aorta. For each patient, the data sets that were reconstructed with IR 3D mild, standard, strong and combined FBP/QDS were simultaneously loaded, and the ROIs were copied from one of the four datasets into the remaining three datasets. As a consequence, all the ROIs were located at exact the same spatial position in the four datasets.

### Analysis of the contour sharpness

**Fig 3:**
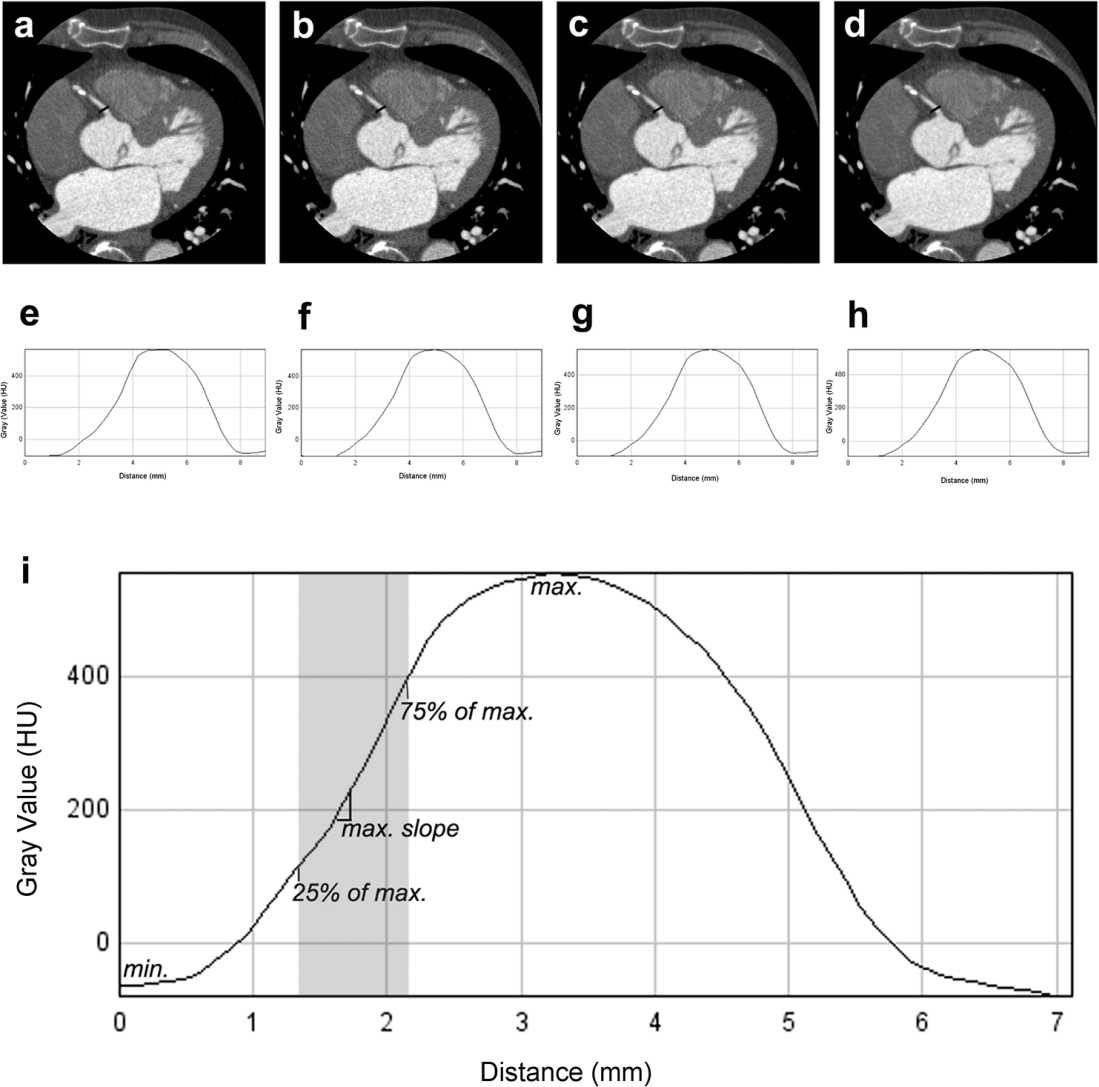
Analysis of contour sharpness. The DICOM images were interpolated by Vitrea fx. The measurement is based on a straight line that is placed orthogonal to the vessels course connecting the epicardial tissue on both sides of the vessel at the example of RCA (**a-d**); **e-h**: gray values in HU along the line above the vessel for the analysis of contour sharpness; see **S5 Table** for statistical results; **a/e:** combined filtered back projection and quantum denoising filtering software; **b/f:** adaptive iterative dose reduction three-dimensional (AIDR 3D) mild; **c/g:** AIDR 3D standard; **d/h:** AIDR 3D strong; **i:** The analysis of contour sharpness is based on the difference between 25% and 75% of the maximal gray value (max.) and the maximal slope of gray values between two pixels in percent of the difference between minimal (min.) and maximal density.

The methods of the contour sharpness analysis have already been published [26]. We performed the measurement by gathering two different parameters: 1) analysis of the distance between 25% and 75% of the maximal grey value in mm and 2) evaluation of the maximal slope in the contour in percent that conformed to the quotient of the maximal difference of grey values between two contiguous pixels and the difference between minimal and maximal grey value in the measurement (**Fig 3**). The measurement of the contour sharpness was performed only in the 4 proximal measurement points in the vessel lumen (**Fig 3**) and the relevant area in the proximal vessel was slanted in the coronal and sagittal slices along the vessels course. A screenshot was created for the four simultaneously loaded, equally slanted images of the datasets at the same slice position. The resulting images were exported from Vitrea fx as DICOM formats. Orthogonally to the vessels course, a straight line was placed in the image connecting the epicardial tissues on both sides of the vessel for the analysis of the contour sharpness without integrating calcified, non-calcified plaques or stents by using ImageJ Version 1.4 (**Fig 3**). By loading the images being reconstructed with FBP/QDS, IR 3D mild, IR 3D standard and IR 3D strong simultaneously, the measurements with ImageJ were performed at the same spatial position for the four reconstructions.

### Statistical analysis

The values are given as arithmetic mean (standard deviation), if not defined otherwise. First, Kolmogorov-Smirnov test was performed to test normal distribution. A p-value of ≤0.05 was considered to indicate statistical significance. An overall analysis was performed with the Repeated Measures ANOVA testing each measurement point and the four reconstructions as dependent variable. Only if p-value was ≤0.05 that was defined as statistical significant, the ANOVA test was separately conducted for each measurement point over the four reconstructions. If the p-value was ≤0.05, the single tests were pairwise performed for each measurement point and the separate reconstructions by using the t-test for dependent variables. The significance level was automatically adapted by SPSS version 20 for the multiple testing with the following 6 single tests by using Bonferroni correction: FBP/QDS-IR 3D mild, FBP/QDS-IR 3D standard, FBP/QDS-IR 3D strong, IR 3D mild-IR 3D standard, IR 3D mild-IR 3D strong, IR 3D standard-IR 3D strong. Dependent on the number of measurement points for each variable, a further correction of the significance level was manually performed by using Bonferroni correction: for the signal and noise measurements, a p-value ≤0.002 was considered statistical significant (21 measurement points), for the SNR and the CNR a p-value ≤0.005 was defined as statistical significant (10 measurement points) and the p-value for the contour sharpness was corrected to a value of ≤0.01 (4 measurement points). The influence of the vessel segments was tested over all 30 patients by ANOVAs test, comparing the relative SNR and CNR differences of the three analysed vessel segments for RCA, LAD and LCX. These relative mean differences were calculated as the difference between each IR 3D level value and the FBP value divided by the FBP value. The means for each vessel segment were calculated and the relative deviation from the means over all vessel segments is illustrated in Blant-Altman Plots for SNR and CNR of each IR 3D level.

## Results

### Patient collective

We included 30 patients with a male-to-female ratio of 1:1 (15 females, 15 males; **Table 1**) and a mean age of 64 years (64.1 ± 9.1 years). The patients were pre-obese with a mean BMI of 29 kg/m² (28.8 ± 4.0 kg/m²). During the CT examination the average HR was <60 bpm (56.2 ± 7.3 bpm). By using a tube voltage of 120 kV the average tube current was 342 mA (341.7 ± 60.0 mA). Mean radiation dose of CTA was 3.2 mSv (3.2 ± 2.6 mSv). In this image quality substudy no measurement was excluded from the analysis, and all measurement points were classified as evaluable.

**Table 1:** Characteristics of the 30 Patients.

Feature			
Age		64.1	±9.1 years
Sex	Female	15	(50%)
	Male	15	(50%)
Abdominal circumference		103.2	±11.8 cm
Height		168.4	±8.7 cm
Weight		81.9	±13.8 kg
BMI		28.8	±4.0 kg/m²
Systolic blood pressure		132.0	±11.3 mmHg
Diastolic blood pressure		79.3	±8.1 mmHg
Arterial hypertension		28	(93%)
Hyperlipidaemia		23	(77%)
Cardiac insufficiency		5	(17%)
Myocardial infarction ^a^		15	(50%)
Smoking		6	(20%)
Heart rate during scan		56.2	±7.3 bpm
Oral beta-blockers		72.5	±41.2 mg
Intravenous beta-blockers		60	±112.5 mg
Tube current		341.7	±60.0 mA
Radiation dose		3.2	±2.6 mSv

Values are given as arithmetic mean ± standard deviation (SD) or number of patients (%)

^a^ Myocardial infarction dated back more than 48 hours

Mean age was 64 years. The average HR was <60 bpm. 25 patients received oral administration of approximately 73 mg atenolol, and 8 of these patients received additional intravenous administration of 60 mg esmolol. Average tube current was 342 mA with mean radiation dose of 3 mSv for CTA.

### Signal

**Fig 4:**
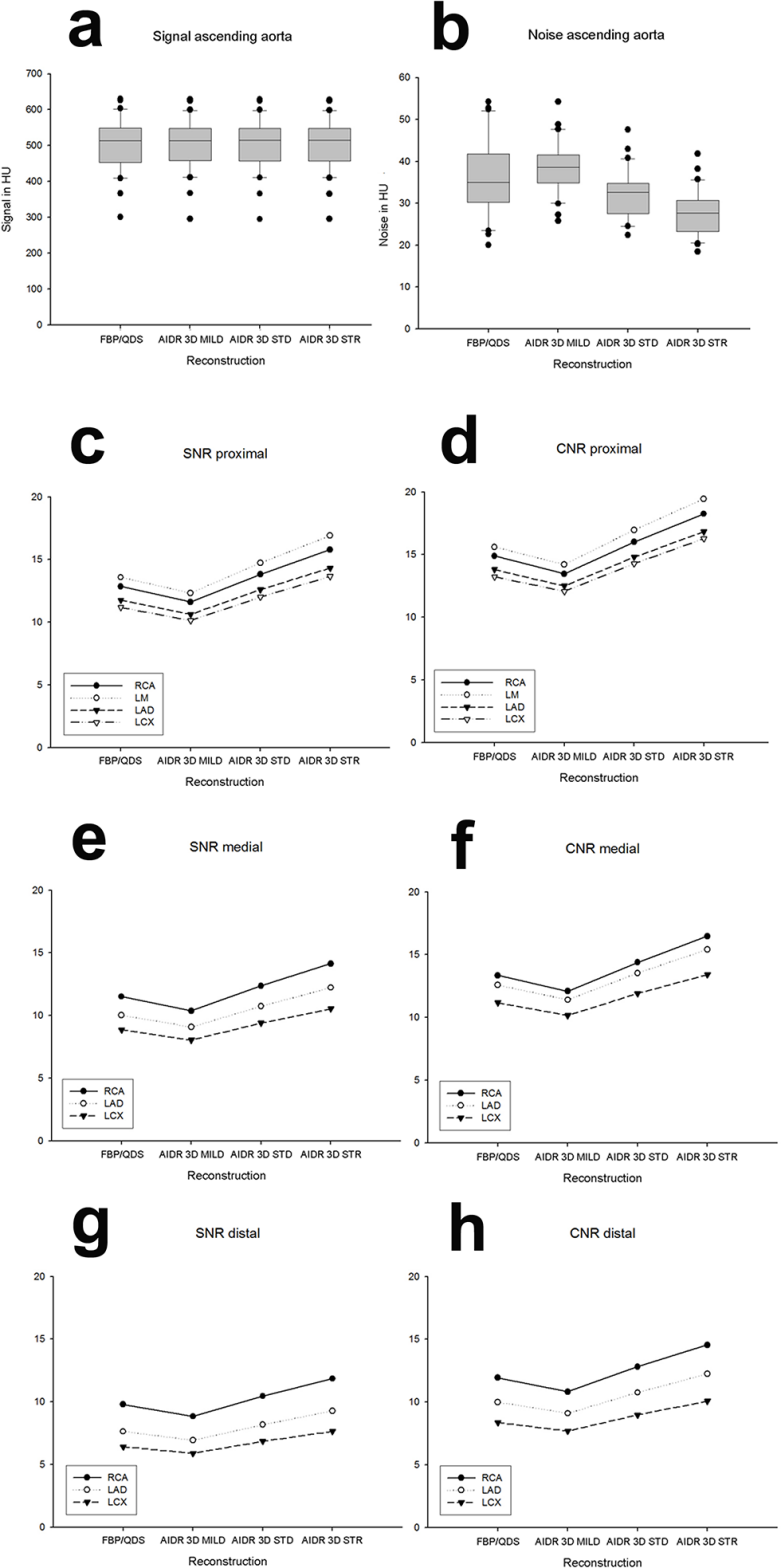
Comparison of quantitative image quality parameters. Signal (**a**) and noise (**b**) of the reconstructions FBP/QDS, AIDR 3D mild, AIDR 3D standard (STD) and AIDR 3D strong (STR) in the ascending aorta measurement point; **c/e/g:** signal-noise-ratio (SNR); **d/f/h:** contrast-noise-ratio (CNR); results in the proximal (**c/d**), mid (**e/f**) and distal measurement points (**g/h**); the same results were found for proximal, medial and distal coronary segments, see **S2–S4 Tables** for statistical results.

The comparison of the signal intensity in the vessel lumen and the surrounding tissue showed no significant difference between FBP/QDS and IR 3D mild (**S2 Table**; **Fig 4**). In the vessel lumen signal intensity was decreased for IR 3D standard in comparison to IR 3D mild by 2% and for IR 3D strong by 2% (at 10 of 11 measurement points p<0.001), while in the surrounding tissue the comparison of signal between IR 3D mild and IR 3D standard (p≥0.007 at 10/10 measurement points), and also for IR 3D strong versus IR 3D standard (p≥0.018 at 7/10 measurement points) showed no statistical significant differences (**S2 Table**).

### Noise

Half of the measurement points in the surrounding tissue showed an increase of noise for IR 3D mild compared to FBP/QDS (p≤0.002; **S3 Table**; **Table 2**). The remaining half of the measurement points in the surrounding tissue, and the 11 measurement points in the vessel lumen showed the same tendency, but without statistical significance after Bonferroni correction (p n.s.; **Fig 4**). Noise was higher for IR 3D mild against IR 3D standard (p<0.001 at 21 measurement points) and also for IR 3D standard compared to IR 3D strong (p≤0.001 at 20/21 measurement points). In the ascending aorta noise increased for IR 3D mild compared to FBP/QDS by 8.6 ± 11.7%, and, noise decreased for IR 3D standard in comparison to FBP/QDS by 9.6 ± 9.0%, and by 21.7 ± 7.8% with IR 3D strong as compared to FBP/QDS.

**Table 2:** Comparison of signal, noise, SNR and CNR between the reconstructions FBP/QDS, AIDR 3D mild, AIDR 3D standard and AIDR 3D strong.

									ANOVA	t-test					
	FBP/QDS		MILD		STD		STR		p	p1	p2	p3	p4	p5	p6
Signal	155.3	(53.7)	155.0	(53.5)	152.5	(53.4)	149.9	(53.3)	<0.001	1.000	<0.001	<0.001	<0.001	<0.001	<0.001
Noise	32.8	(9.3)	34.5	(8.9)	29.8	(8.3)	27.0	(7.8)	<0.001	<0.001	<0.001	<0.001	<0.001	<0.001	<0.001
SNR	10.4	(3.0)	9.4	(3.0)	11.1	(3.7)	12.6	(4.4)	<0.001	0.001	0.001	<0.001	<0.001	<0.001	<0.001
CNR	12.5	(4.3)	11.4	(3.1)	13.5	(3.9)	15.3	(4.7)	<0.001	0.002	<0.001	<0.001	<0.001	<0.001	<0.001

Values are given in arithmetic mean (SD); reconstruction with filtered back projection/ quantum denoising filtering system (**FBP/QDS)**, adaptive iterative dose reduction (AIDR) 3D mild (**MILD**), standard (**STD**) and strong (**STR**); The values of all measurement points are summarised; First, Repeated Measures ANOVA overall analysis including every measurement point as dependent variable showed p<0.001 (p ANOVA) for signal, noise, SNR and also CNR. Subsequently, t-test was used as single test for the 4 quantitative parameters. Bonferroni correction was automatically performed for the multiple testing with 6 possibilities: **p1** (FBP/QDS-AIDR 3D mild), **p2** (FBP/QDS-AIDR 3D standard), **p3** (FBP/QDS-AIDR 3D strong), **p4** (AIDR 3D mild-AIDR 3D standard), **p5** (AIDR 3D mild-AIDR 3D strong), **p6** (AIDR 3D standard-AIDR 3D strong); **signal** presents the density in Hounsfield Units; **noise** is SD of the signal; **SNR** = signal in the vessel measurement point/noise in the aorta ascendens measurement point; **CNR** = (signal in the surrounding tissue of the vessel measurement point—signal in the vessel measurement point)/noise in the aorta ascendens measurement point.

### SNR and CNR

Eight of 10 measurement points were each characterised by a reduction of both SNR and CNR for IR 3D mild against FBP/QDS (SNR reduction of 10% and CNR reduction of 9%), all 10 measurement points showed an increase of SNR and CNR by using IR 3D standard in comparison to IR 3D mild (SNR increase of 19% and CNR increase of 18%) and also for IR 3D strong as compared to the IR 3D standard (p≤0.004, respectively; **Fig 4**; **S4 Table**; **Table 2**; SNR and CNR increase of 14%). The two measurement points that showed no statistical significance in the comparison between IR 3D mild and FBP/QDS were located in the distal LCX and LAD (p>0.005).

**Fig 5:**
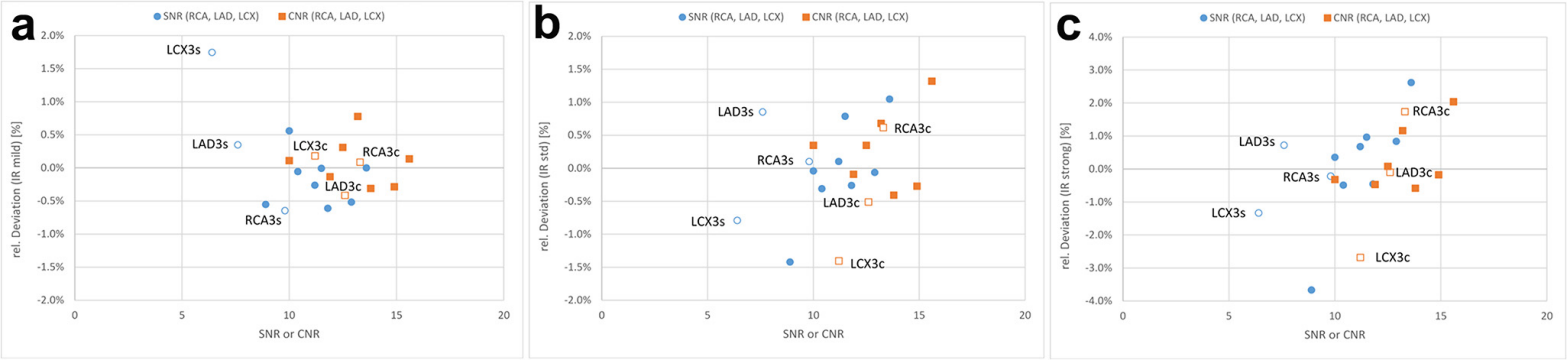
Bland Altman plots of SNR and CNR. The percentage relative deviation of SNR and CNR of each measurement point from the mean relative deviation for that particular IR level from FBP/QDS is plotted against the corresponding FBP/QDS measurement. Distal segments (RCA3, LAD3 and LCX3) are shown as open marked dots. Mainly, the relative deviation of SNR and CNR in distal segments is not significantly different compared to that of proximal segments; **a**: AIDR mild; relative deviation is less than 2% of the mean relative deviation of all segments, **b**: AIDR standard; Relative deviation is less than 1.5% of the mean relative deviation of all segments, **c**: AIDR strong; Relative deviation is less than 4% of the mean relative deviation of all segments.

Testing the 3 vessel segments over all 30 patients due to the relative SNR, the significances were determined for IR 3D mild p>0.754 in RCA, p>0.114 in LAD, p>0.634 in LCX; for IR 3D standard p>0.349 in RCA, p>0.459 in LAD, p>0.371 in LCX and for IR 3D strong p>0.011 in RCA, p>0.923 in LAD and p>0.012 in LCX and due to the relative CNR the significances were determined for IR 3D mild p>0.451 in RCA, p>0.032 in LAD, p>0.605 in LCX; for IR 3D standard p>0.255 in RCA, p>0.222 in LAD, p>0.432 in LCX and for IR 3D strong p>0.004 in RCA, p>0.340 in LAD and p>0.020 in LCX. In summary slight differences were found, using IR 3D mild for CNR in LAD and using IR 3D strong for SNR and CNR in RCA and LCX. With IR 3D standard no differences in SNR or CNR were found. The relative deviations from the FBP means over all analysed vessel segments, as illustrated in Blant-Altman Plots of **Fig 5**are confirming visually these non-significant differences of the distal vessels (open marked dots) compared with the middle and proximal vessels (filled dots) of RCA, LAD, LCX, LM and aorta using the three IR 3D levels.

### Contour sharpness

**Fig 6:**
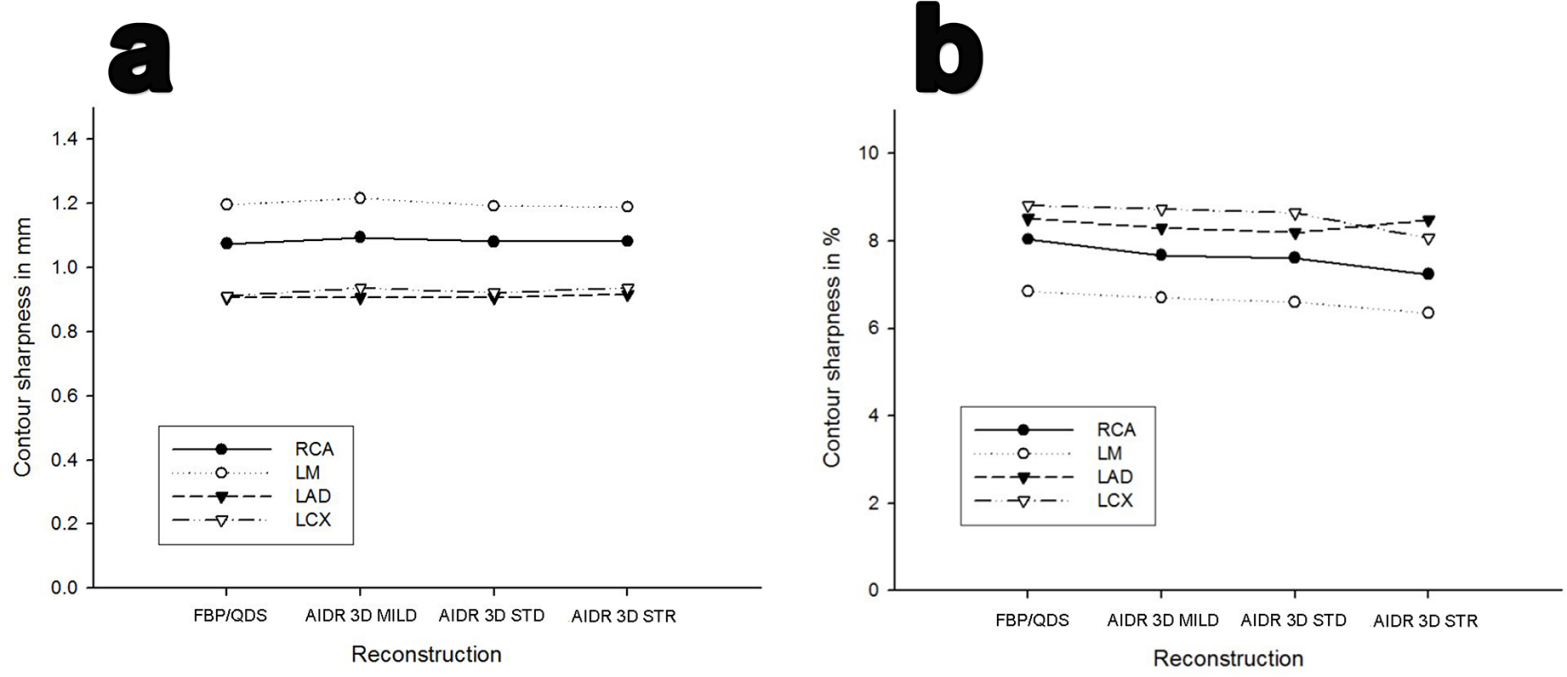
Comparison of contour sharpness. No significant difference was found for the contour sharpness based on the difference between 25% and 75% of the maximal gray value (**a**) and the maximal slope of gray values between two pixels in percent of the difference between minimal and maximal density (**b**); comparison between FBP/QDS, AIDR 3D mild, AIDR 3D standard (STD) and AIDR 3D strong (STR).

The intraindividual comparison between the 4 reconstructions showed no significant difference regarding the contour sharpness based on the distance between 25% and 75% of the maximal grey value (**S5 Table**; **Fig 6**; p>0.08). The comparison of the maximal slope of grey values in the contour showed a deterioration by using the IR 3D strong as compared to the FBP/QDS at the LCX measurement point (p = 0.004). The remaining single comparisons showed no statistical significance (**S5 Table**).

## Discussion

The use of AIDR 3D strong as compared to FBP/QDS in standard dose coronary CTA improved the following image quality parameters: strongest noise reduction, highest values for SNR and CNR. Despite the expectation of reduced contour sharpness for AIDR 3D, there was no disadvantage in comparison to FBP/QDS. The use of AIDR 3D mild resulted in similar image quality as compared to the combination of FBP/QDS when evaluating these parameters at a noise level adapted for FBP/QDS.

Coronary CTA is able to detect significant coronary stenosis with a high diagnostic accuracy [1]. In patients with acute coronary syndrome and a low to intermediate likelihood, CTA is a safe and efficient alternative to the CCA [27]. With due regard to the diagnostic accuracy and cost-effectiveness in comparison to CCA, CTA may represent an first-step-diagnostic alternative in those patients [27].

In patients with known CAD coronary stents may decrease the diagnostic accuracy [23]. Instead of the FBP reconstruction also IR can be used in those patients to improve the image quality [21].

The major components of AIDR 3D include noise reduction processing in the projection data domain and iterations in the reconstruction domain [15]. Since the former is most efficient at high noise levels, noise reduction by AIDR 3D is most effective at low mAs settings (<100 mAs). The strength of the noise reduction processing in the projection data domain and the number of iterations in the image data domain are increased from AIDR 3D mild to AIDR 3D strong, resulting in highest noise reduction with AIDR 3D strong. For each application, the algorithm has been developed using as a reference FBP reconstructions at noise levels that are routine for FBP/QDS. At corresponding standard dose levels, SD values of images reconstructed with AIDR 3D standard should be similar to those of FBP/QDS reconstructions. In contrast, when data acquisition is performed at considerably lower radiation dose, lower SD values are expected for AIDR 3D as compared to FBP/QDS. Moreover, due to a nonlinear relationship, the difference in noise levels between AIDR 3D and FBP/QDS will increase with decreasing radiation exposure.

In our current study, we performed a systematic evaluation of the effect of the different levels of AIDR 3D on image quality of coronary CTA studies acquired at tube current levels currently routine for FBP/QDS. Increased noise reduction was observed with each level of AIDR 3D from mild to strong. As expected, the use of AIDR 3D mild at these high mA levels did not improve image quality parameters as compared to FBP/QDS. Nevertheless, the use of AIDR 3D strong within this particular mA range still provided improvement in image quality. This was reflected by the strongest noise reduction as well as highest values for SNR and CNR, all significantly improved as compared to FBP/QDS. No effect of AIDR 3D on contour sharpness was observed in this study. Slight differences were found for SNR and CNR between distal and proximal coronary segments, because the intensively smoothing in AIDR mild and advanced enhancement in AIDR strong influences the CT numbers in the proximal segments more intensively than in in the mid and distal segments.

Other studies that compared the image quality between iterative reconstructions and FBP showed similar results: the raw data based HIR is characterised by higher image quality parameters based on higher values for both SNR and CNR, while noise is reduced without degrading signal intensity values [11, 28]. SAFIRE shows a reduction of noise and also higher subjective image quality compared to FBP [29]. In recent studies that compare AIDR 3D to FBP, noise was reduced, while SNR, CNR and subjective interpretation capacity were improved for AIDR 3D [30–33].

A recent study of Yang et al. that was performed as an intraindividual comparison, compared the image quality of five iteration levels of the raw data based SAFIRE as compared to the conventional FBP in a collective including 304 patients by using dual source CT [17]. This study showed an association between increase of the filter strength and reduction of noise, while CNR and SNR were improved without degrading signal intensity values. Our analysis showed the same results for the different levels of the AIDR 3D, with exception of mild levels. The usage of different iterative reconstruction methods could be a relevant factor for the discrepancy of the results of Yang et al. compared to that of our analysis. Additionally, in our study higher tube current was used as compared to the study from Yang et al. (43 ± 12 mA versus 342 ± 60 mA) and the patients in our study showed a higher BMI that is associated with an increase of noise, in general (23 ± 3 kg/m² versus 29 ± 4 kg/m²).

Despite the value of subjective image quality analysis for evaluating the diagnostic accuracy, we performed a systematic and objective evaluation that shows the advantage to avoid potential observer bias. Based on recent reviews [34, 35], the quantification of objective image quality parameters is commonly performed by gathering SNR, CNR and noise (20 min per patient). In our analysis, for the first time a differentiation between proximal, mid and distal coronary segments was performed for the analysis of the CNR and the SNR (60 min per patient). Additionally, the evaluation of the contour sharpness was used to scientifically analyse the image quality (2.5 h per patient) based on a recent work that has already been published in PLoS ONE [26].

The small patient collective is a relevant limitation of our study. The comparison of the contour sharpness showed no statistical significant difference in our analysis. Due to the small patient collective we decided to perform an intraindividual comparison instead of a randomised trial that may show differences in one patient at the same position in the coronary segment, and not only between groups. Thus, our patients underwent the CT by using the same scanning conditions for both, AIDR 3D and FBP. Performing the AIDR 3D with lower radiation dose might have influenced the contour sharpness. In our analysis, FBP reconstruction was combined with QDS for additional noise reduction. Due to this difference in reference standard, comparison to recent studies is only partially possible. If ROIs are placed manually into distal coronary segments with a small diameter, the vessel wall could be included in the measurement and, as a result, the statistical noise is influenced by the profile of CT number and noise increases. In addition, we summarized the LM and proximal LAD, LCX and RCA to proximal measurement points though, in general, the LM vessel size is superior to that of LAD, LCX and RCA. While in our analysis there was no difference of SNR and CNR between proximal and distal segments, the image quality might me influenced by small changes of the vessel size. Although coronary CTA may be used to specify the different plaque entities [36], we did not include a plaque analysis in our manuscript. Finally, our standard scanning conditions were above 100 mAs. Even for standard clinical acquisition protocols of coronary CTA that acquire with mAs values above 100 mAs, AIDR 3D remains effective compared to original FBP to reduce SD values and improve the image quality, however the effect is less outspoken when compared to the former QDS+ technique of Toshiba. As the noise reduction effects of these iterative reconstruction techniques are expected more pronounced at lower mAs values, it will be important to explore coronary CTA protocols at lower mAs in future studies.

In our analysis, the image quality was optimised by using AIDR 3D strong that showed highest values for both SNR and CNR, while reducing noise most efficiently. Accordingly, when used at the same radiation dose, reconstructing with AIDR 3D strong will result in improved image quality as compared to FBP/QDS. Alternatively, if the aim is to have similar image quality, radiation dose can be reduced using AIDR 3D strong as compared to FBP/QDS. In contrast, the use of AIDR 3D mild resulted in similar image quality as compared to the combination of FBP/QDS when evaluating these parameters at a noise level adapted for FBP/QDS and thus may not provide additional benefit when applied in these scanning conditions. Mainly, in the distal segments of the RCA, LAD and LCX vessels the relative deviations in CNR/SNR are nearly similar to changes in the middle and proximal vessels.

## Supporting Information

S1 Checklist TREND checklist. (PDF)

S1 Protocol Study Protocol. (PDF)

S1 Table Image quality analysis. (XLSX)

S2 Table Analysis of the signal. (DOCX)

S3 Table Analysis of the noise. (DOCX)

S4 Table Analysis of the signal-noise-ratio (SNR) and contrast-noise-ratio (CNR). (DOCX)

S5 Table Analysis of the contour sharpness. (DOCX)

## References

[1] Dewey M, Zimmermann E, Deissenrieder F, Laule M, Dübel HP, Schlattmann P, et al Noninvasive coronary angiography by 320-row computed tomography with lower radiation exposure and maintained diagnostic accuracy: comparison of results with cardiac catheterization in a head-to-head pilot investigation. Circulation. 2009;120(10):867–75. 10.1161/CIRCULATIONAHA.109.859280 . 19704093

[2] Pelliccia F, Pasceri V, Evangelista A, Pergolini A, Barillà F, Viceconte N, et al Diagnostic accuracy of 320-row computed tomography as compared with invasive coronary angiography in unselected, consecutive patients with suspected coronary artery disease. Int J Cardiovasc Imaging. 2013;29(2):443–52. 10.1007/s10554-012-0095-4 . 22806317

[3] Kubo T, Lin PJ, Stiller W, Takahashi M, Kauczor HU, Ohno Y, et al Radiation dose reduction in chest CT: a review. AJR Am J Roentgenol. 2008;190(2):335–43. 10.2214/AJR.07.2556 . 18212218

[4] Sun K, Han RJ, Ma LJ, Wang LJ, Li LG, Chen JH. Prospectively electrocardiogram-gated high-pitch spiral acquisition mode dual-source CT coronary angiography in patients with high heart rates: comparison with retrospective electrocardiogram-gated spiral acquisition mode. Korean J Radiol. 2012;13(6):684–93. 10.3348/kjr.2012.13.6.684 23118566 PMC3484288

[5] Paul NS, Blobel J, Prezelj E, Burey P, Ursani A, Menezes RJ, et al The reduction of image noise and streak artifact in the thoracic inlet during low dose and ultra-low dose thoracic CT. Phys Med Biol. 2010;55(5):1363–80. 10.1088/0031-9155/55/5/007 . 20145292

[6] Eisentopf J, Achenbach S, Ulzheimer S, Layritz C, Wuest W, May M, et al Low-Dose Dual-Source CT Angiography With Iterative Reconstruction for Coronary Artery Stent Evaluation. JACC Cardiovasc Imaging. 2013;6(4):458–65. 10.1016/j.jcmg.2012.10.023 . 23498678

[7] Scheffel H, Stolzmann P, Schlett CL, Engel LC, Major GP, Károlyi M, et al Coronary artery plaques: cardiac CT with model-based and adaptive-statistical iterative reconstruction technique. Eur J Radiol. 2012;81(3):e363–9. 10.1016/j.ejrad.2011.11.051 . 22197733

[8] Renker M, Nance JW, Schoepf UJ, O'Brien TX, Zwerner PL, Meyer M, et al Evaluation of heavily calcified vessels with coronary CT angiography: comparison of iterative and filtered back projection image reconstruction. Radiology. 2011;260(2):390–9. 10.1148/radiol.11103574 . 21693660

[9] Silva AC, Lawder HJ, Hara A, Kujak J, Pavlicek W. Innovations in CT dose reduction strategy: application of the adaptive statistical iterative reconstruction algorithm. AJR Am J Roentgenol. 2010;194(1):191–9. 10.2214/AJR.09.2953 . 20028923

[10] Bittencourt MS, Schmidt B, Seltmann M, Muschiol G, Ropers D, Daniel WG, et al Iterative reconstruction in image space (IRIS) in cardiac computed tomography: initial experience. Int J Cardiovasc Imaging. 2011;27(7):1081–7. 10.1007/s10554-010-9756-3 . 21120612

[11] Oda S, Utsunomiya D, Funama Y, Yonenaga K, Namimoto T, Nakaura T, et al A hybrid iterative reconstruction algorithm that improves the image quality of low-tube-voltage coronary CT angiography. AJR Am J Roentgenol. 2012;198(5):1126–31. 10.2214/AJR.11.7117 . 22528903

[12] Wang R, Schoepf UJ, Wu R, Gibbs KP, Yu W, Li M, et al CT coronary angiography: image quality with sinogram-affirmed iterative reconstruction compared with filtered back-projection. Clin Radiol. 2013;68(3):272–8. 10.1016/j.crad.2012.08.007 . 22981731

[13] Ebersberger U, Tricarico F, Schoepf UJ, Blanke P, Spears JR, Rowe GW, et al CT evaluation of coronary artery stents with iterative image reconstruction: improvements in image quality and potential for radiation dose reduction. Eur Radiol. 2013;23(1):125–32. 10.1007/s00330-012-2580-5 . 22777622

[14] Tomizawa N, Nojo T, Akahane M, Torigoe R, Kiryu S, Ohtomo K. AdaptiveIterative Dose Reduction in coronary CT angiography using 320-row CT: assessment of radiation dose reduction and image quality. J Cardiovasc Comput Tomogr. 2012;6(5):318–24. 10.1016/j.jcct.2012.02.009 . 22981854

[15] Blobel J, Mews J, Schuijf JD, Overlaet W. Determining the radiation dose reduction potential for coronary calcium scanning with computed tomography: an anthropomorphic phantom study comparing filtered backprojection and the adaptive iterative dose reduction algorithm for image reconstruction. Invest Radiol. 2013;48(12):857–62. 10.1097/RLI.0b013e31829e3932 . 23917328

[16] Chen MY, Steigner ML, Leung SW, Kumamaru KK, Schultz K, Mather RT, et al Simulated 50% radiation dose reduction in coronary CT angiography using adaptive iterative dose reduction in three-dimensions (AIDR3D). Int J Cardiovasc Imaging. 2013;29(5):1167–75. 10.1007/s10554-013-0190-1 23404384 PMC3701132

[17] Yang WJ, Yan FH, Liu B, Pang LF, Hou L, Zhang H, et al Can sinogram-affirmed iterative (SAFIRE) reconstruction improve imaging quality on low-dose lung CT screening compared with traditional filtered back projection (FBP) reconstruction? J Comput Assist Tomogr. 2013;37(2):301–5. 10.1097/RCT.0b013e31827b8c66 . 23493224

[18] Leipsic J, Labounty TM, Heilbron B, Min JK, Mancini GB, Lin FY, et al Adaptive statistical iterative reconstruction: assessment of image noise and image quality in coronary CT angiography. AJR Am J Roentgenol. 2010;195(3):649–54. 10.2214/AJR.10.4285 . 20729442

[19] Kligerman S, Mehta D, Farnadesh M, Jeudy J, Olsen K, White C. Use of a hybrid iterative reconstruction technique to reduce image noise and improve image quality in obese patients undergoing computed tomographic pulmonary angiography. J Thorac Imaging. 2013;28(1):49–59. 10.1097/RTI.0b013e31825412b2 . 22576762

[20] Willemink MJ, Borstlap J, Takx RA, Schilham AM, Leiner T, Budde RP, et al The effects of computed tomography with iterative reconstruction on solid pulmonary nodule volume quantification. PLoS One. 2013;8(2):e58053 10.1371/journal.pone.0058053 23460924 PMC3584042

[21] Gebhard C, Fiechter M, Fuchs TA, Stehli J, Müller E, Stähli BE, et al Coronary artery stents: influence of adaptive statistical iterative reconstruction on image quality using 64-HDCT. Eur Heart J Cardiovasc Imaging. 2013;14(10):969–77. 10.1093/ehjci/jet013 . 23428650

[22] Des Jarlais DC, Lyles C, Crepaz N, Group T. Improving the reporting quality of nonrandomized evaluations of behavioral and public health interventions: the TREND statement. Am J Public Health. 2004;94(3):361–6. 14998794 10.2105/ajph.94.3.361 PMC1448256

[23] Rief M, Zimmermann E, Stenzel F, Martus P, Stangl K, Greupner J, et al Computed tomography angiography and myocardial computed tomography perfusion in patients with coronary stents: prospective intraindividual comparison with conventional coronary angiography. J Am Coll Cardiol. 2013;62(16):1476–85. 10.1016/j.jacc.2013.03.088 . 23792193

[24] Rief M, Stenzel F, Kranz A, Schlattmann P, Dewey M. Time efficiency and diagnostic accuracy of new automated myocardial perfusion analysis software in 320-row CT cardiac imaging. Korean J Radiol. 2013;14(1):21–9. 10.3348/kjr.2013.14.1.21 23323027 PMC3542299

[25] Hoffmann MH, Lessick J, Manzke R, Schmid FT, Gershin E, Boll DT, et al Automatic determination of minimal cardiac motion phases for computed tomography imaging: initial experience. Eur Radiol. 2006;16(2):365–73. 10.1007/s00330-005-2849-z . 16021450

[26] Enders J, Rief M, Zimmermann E, Asbach P, Diederichs G, Wetz C, et al High-field open versus short-bore magnetic resonance imaging of the spine: a randomized controlled comparison of image quality. PLoS One. 2013;8(12):e83427 10.1371/journal.pone.0083427 24391767 PMC3877023

[27] D'Ascenzo F, Cerrato E, Biondi-Zoccai G, Omedè P, Sciuto F, Presutti DG, et al Coronary computed tomographic angiography for detection of coronary artery disease in patients presenting to the emergency department with chest pain: a meta-analysis of randomized clinical trials. Eur Heart J Cardiovasc Imaging. 2013;14(8):782–9. 10.1093/ehjci/jes287 . 23221314

[28] Utsunomiya D, Weigold WG, Weissman G, Taylor AJ. Effect of hybrid iterative reconstruction technique on quantitative and qualitative image analysis at 256-slice prospective gating cardiac CT. Eur Radiol. 2012;22(6):1287–94. 10.1007/s00330-011-2361-6 . 22200900

[29] Moscariello A, Takx RA, Schoepf UJ, Renker M, Zwerner PL, O'Brien TX, et al Coronary CT angiography: image quality, diagnostic accuracy, and potential for radiation dose reduction using a novel iterative image reconstruction technique-comparison with traditional filtered back projection. Eur Radiol. 2011;21(10):2130–8. 10.1007/s00330-011-2164-9 . 21611758

[30] Tatsugami F, Matsuki M, Nakai G, Inada Y, Kanazawa S, Takeda Y, et al The effect of adaptive iterative dose reduction on image quality in 320-detector row CT coronary angiography. Br J Radiol. 2012;85(1016):e378–82. 10.1259/bjr/10084599 22253355 PMC3495581

[31] Gervaise A, Osemont B, Lecocq S, Noel A, Micard E, Felblinger J, et al CT image quality improvement using Adaptive Iterative Dose Reduction with wide-volume acquisition on 320-detector CT. Eur Radiol. 2012;22(2):295–301. 10.1007/s00330-011-2271-7 . 21927791

[32] Yamada Y, Jinzaki M, Hosokawa T, Tanami Y, Sugiura H, Abe T, et al Dose reduction in chest CT: Comparison of the adaptive iterative dose reduction 3D, adaptive iterative dose reduction, and filtered back projection reconstruction techniques. Eur J Radiol. 2012;81(12):4185–95. 10.1016/j.ejrad.2012.07.013 . 22883532

[33] Yoo RE, Park EA, Lee W, Shim H, Kim YK, Chung JW, et al Image quality of adaptive iterative dose reduction 3D of coronary CT angiography of 640-slice CT: comparison with filtered back-projection. Int J Cardiovasc Imaging. 2013;29(3):669–76. 10.1007/s10554-012-0113-6 . 22923280

[34] Willemink MJ, de Jong PA, Leiner T, de Heer LM, Nievelstein RA, Budde RP, et al Iterative reconstruction techniques for computed tomography Part 1: technical principles. Eur Radiol. 2013;23(6):1623–31. 10.1007/s00330-012-2765-y . 23314600

[35] Willemink MJ, Leiner T, de Jong PA, de Heer LM, Nievelstein RA, Schilham AM, et al Iterative reconstruction techniques for computed tomography part 2: initial results in dose reduction and image quality. Eur Radiol. 2013;23(6):1632–42. 10.1007/s00330-012-2764-z . 23322411

[36] Post WS, Budoff M, Kingsley L, Palella FJ, Witt MD, Li X, et al Associations between HIV infection and subclinical coronary atherosclerosis. Ann Intern Med. 2014;160(7):458–67. 10.7326/M13-1754 24687069 PMC4143766

