# Supplementary material for: The Impact of Different Levels of Adaptive Iterative Dose Reduction 3D on Image Quality of 320-Row Coronary CT Angiography: A Clinical Trial
Source: PLoS One. 2015 May 6;10(5):e0125943. doi: 10.1371/journal.pone.0125943 (PMC4422621; doi:10.1371/journal.pone.0125943)
Supplement: S1 Protocol — (PDF) [file pone.0125943.s002.pdf]

CLINICAL STUDY PROTOCOL

Coronary Artery Stent Evaluation With 320-slice Computed Tomography

The “CARS 320” Study

A Prospective, Comparative, Intention-to-diagnose Trial in Patients with Suspected Coronary In-stent Restenosis

Final Protocol Version, dated 17 June 2008

Charité
Humboldt-Universität zu Berlin
Confidential

Table of Contents

1 SUMMARY......3

2 GENERAL INFORMATION......4

Title......4
Organisation......4
Collaborations......4

3 BACKGROUND......6

Coronary Artery Stents......6
Economic Considerations......6
Conventional Coronary Angiography......7
Noninvasive MSCT Coronary Angiography (CTA)......7
Implications for the Design of the Present Study......8

4 TRIAL OBJECTIVES......10

Primary Objective......10
Secondary Objectives......10

5 TRIAL DESIGN......12

Number of Participants and Duration......12
Study Design......12
Withdrawal......12

6 SELECTION OF PATIENTS......13

Screening......13
Informed Consent......13
Inclusion Criteria......13
Exclusion Criteria......14
Medical History......15
Physical Examination......15
Electrocardiography......15
Baseline Laboratory Tests......15
Further Baseline Tests......16

7 MSCT......17

MSCT 17
Preparation 17
Scanning 18
Non-cardiac Findings on MSCT 20
8 ASSESSMENT OF EFFICACY 21
Primary Efficacy Endpoints 21
Secondary Efficacy Endpoints 21
9 ASSESSMENT OF SAFETY 22
Adverse Events Monitoring for MSCT 22
Laboratory Test Results 22
Clinical Adverse Events 22
Serious Adverse Events 23
11 STATISTICS 24
12 QUALITY CONTROL AND ASSURANCE 25
Methods Against Bias 25
SOPs 26
13 ETHICS 27
General 27
Time Intervals between Procedures 27
Institutional Review Board 27
Federal Department for Radiation Protection 27
Modification of the Protocol 28
14 PUBLICATION POLICY 29
15 ORGANIZATION 30
16 REFERENCES 31

1 Summary

Coronary artery disease (CAD) is a major public health issue accounting for 20% of all deaths in Germany and affecting 13.2 million persons and causing more than 500,000 deaths in the United States each year. Commonly coronary artery stenoses are treated using stent placement. The follow-up of these stents for detection of in-stent restenoses is currently based on (invasive) conventional coronary angiography as the definite reference standard, which is performed for diagnostic purposes in more than three out of four cases after stent treatment. Over the last years two noninvasive diagnostic tests for CAD diagnosis have been developed - multislice computed tomography (MSCT) and magnetic resonance imaging (MRI). Due to metal-related artifacts MRI is not suited for detection of coronary artery in-stent restenoses. Despite that MSCT is less limited by such artifacts diagnostic accuracy of MSCT for detection of in-stent restenoses has varied greatly among studies using 16- and 64-slice configuration. The primary aim of this study is to demonstrate a per-patient diagnostic accuracy of combined 320-slice CT for coronary angiography (CTA) and myocardial perfusion (CTP) significantly greater than 75% at an expected per-patient diagnostic accuracy of 87%. For this purpose at a power of 80% and an alpha level of 0.05 altogether 90 patients will have to be recruited.

2 General Information

Title

Coronary Artery Stent Evaluation With 320-slice Computed Tomography ("CArS 320 Study")

Organisation

Name	Title/Designation	Contact Numbers
Charité, Berlin Humboldt-Universität zu Berlin		
Marc Dewey, MD	Coordinating Investigator	Phone: +49 30 4505 27296 or +49 30 4505 27031<br>Fax: +49 30 4505 27996<br>E-mail: marc.dewey@charite.de
Peter Martus, PhD	Trial Statistician	Phone: +49 30 4505 62161 or +49 30 8445 2091/92<br>Fax: +49 30 4505 62972<br>E-mail: peter.martus@charite.de
Karl Stangl, MD	Investigator, Cardiology	karl.stangl@charite.de
Elke Zimmermann, MD	Co-Investigator, Radiology	elke.zimmermann@charite.de
Matthias Rief, MD	Co-Investigator, Radiology	matthias.rief@charite.de
Fabian Knebel	Co-Investigator, Cardiology	fabian.knebel@charite.de
Michael Laule, MD	Co-Investigator, Cardiology	michael.laule@charite.de
Peter Schlattmann, PhD	Co-Statistician	peter.schlattmann@charite.de

Collaborations

Name	Title/Designation	Contact Numbers
Charité, Berlin Humboldt-Universität zu Berlin		
Jacqueline Müller-Nordhorn,	Cost-Effectiveness	Phone: +49 30 4505 29026

MD	Evaluation	Fax: +49 30 4505 29902<br>E-mail: jacqueline.mueller-nordhorn@charite.de
Medizinische Hochschule Hannover		
Eva Schönenberger, MD	Patient Acceptance Evaluation	Phone: +49 511 5326 319<br>Fax: +49 511 552366<br>E-mail: schoenenberger.eva@mh-hannover.de
Leiden University Medical Center<br>Koos Geleijns, PhD	Radiation Exposure Comparison	+31 71 526 2993<br>E-mail: k.geleijns@lumc.nl

The trial will be conducted in compliance with the protocol, GCP, and the applicable regulatory requirements.

3 Background

Coronary Artery Stents

Coronary artery disease (CAD) is a major public health issue, for instance in the United States CAD affects 13.2 million persons and is responsible for more than 500,000 deaths each year (American Heart Association. Heart Disease and Stroke Statistics – Update. 2004), while in Germany 20% of all deaths are due to CAD (Gesundheitsbericht). CAD diminishes local, regional, or global blood supply to the heart and is most commonly caused by coronary atherosclerosis. CAD may manifest as angina pectoris, myocardial infarction, sudden cardiac death, or as severe cardiac insufficiency in ischemic cardiomyopathy. Commonly coronary artery stenoses are treated using stent placement. The follow-up of these stents for detection of in-stent restenoses is currently based on (invasive) conventional coronary angiography as the definite reference standard, which is performed for diagnostic purposes in more than three out of four cases after stent treatment. MSCT is a fast and reliable diagnostic tool to noninvasively assess the coronary arteries for stenoses (Hoffmann et al. JAMA 2005, Dewey et al. 2006 Annals of Internal Medicine 2006). Besides detection of stenoses in the native coronary arteries, assessment of coronary artery in-stent restenosis is of increasing clinical importance as the number of catheter-based coronary interventions is on the rise (currently more than 200,000 in Germany per year). Thus, a reliable noninvasive alternative would be an important clinical improvement.

Economic Considerations

From what has been outlined above it is obvious that diagnosis and therapy of CAD are frequent events and thus account for a considerable part of health care reimbursement. The average expenditure for each patient with CAD is approximately € 64,000 (1). With a decreasing mortality and an increasing number of patients since the 1970s, the direct annual expenditure for outpatient and inpatient treatment in Germany was 2.73 billion (1995) and 3.36 billion € (1996) (1). In the US the overall cost of an AMI including acute catheter intervention, cardiac rehabilitation, and subsequent loss of working hours is $73.000 on average (2). The US spent an estimated $111.8 billion on CAD in 2002 (3). Currently, conventional coronary angiography is performed 874,648-times per year at costs of at least 630 € for each examination. With MSCT and costs of approximately 175 € there is the potential to significantly reduce health care costs provided that clinical efficacy in clinical management can be proven (4). Thus, this trial will influence both clinical and economical approaches to diagnosis and management of coronary

artery disease in Germany and other countries.

Conventional Coronary Angiography

Conventional coronary angiography is the reference standard for definitive diagnosis of coronary artery in-stent restenoses (Guideline of the German Cardiac Society. Diagnose und Behandlung der chronischen koronaren Herzerkrankung). According to the latest report of the German Cardiac Society, over 700,000 conventional coronary angiographies were performed in 2003 (5). Approximately three out of four conventional coronary angiographies performed for follow-up of patients with coronary stents are for diagnostic purposes only since relevant lesion can be excluded in these patients. To replace these diagnostic examinations with noninvasive modalities would be worthwhile. Moreover, conventional coronary angiography carries a risk of complications that is not negligible (6). This problem may be overcome by the use of noninvasive coronary angiography.

Noninvasive MSCT Coronary Angiography (CTA)

With the recent multislice CT (MSCT) scanners using submillimeter detector collimation and thus isotropic voxel sizes of down to 0.5*0.35*0.35 mm, reliable detection of coronary artery in-stent restenoses is feasible but has shown rather variable results in several studies comparing MSCT to conventional coronary angiography (**Table 1**). It is noteworthy that in the studies including all patients with coronary stents regardless of internal stent diameter (7-9), a considerable proportion of stents had to be excluded from analysis due to poor image quality. Adequate evaluation with an accuracy suitable for clinical use was possible only for stents with an internal diameter of at least 3.5 mm (7). An only moderate diagnostic accuracy was achieved in those segments that could be evaluated (Table). In contrast to these results, two studies investigating only patients with stents in the left main branch found a fairly high sensitivity of over 90% (10, 11). Two further recent studies investigating stents in all coronary arteries as well as bypass grafts in a total of 64 (12) and 143 patients (13) suggest that adequate assessment of the stent lumen is possible only in about 50% of all stents with an internal diameter of up to 3.0 mm. Therefore, sensitivities and specificities are very poor when all stents are included in the analysis (**Table 1**). Altogether, these results show that current MSCT scanners still have marked limitations in the detection of coronary artery in-stent restenoses. These limitations might be overcome if, in addition to improving spatial and temporal resolution, information on blood flow can be obtained. Myocardial blood flow cannot be assessed with currently available CT scanners because they do not yield functional information. Moreover, small stents cannot be reliably assessed due to their small diameter and beam hardening artefacts. The next generation of 320-

slice scanners currently being developed (14) provide functional information (4D analysis) that allows evaluation of blood flow and therefore might improve the evaluation of coronary stents.

Table 1 **Results of a Systematic Review for the Comparison of MSCT and Conventional Coronary Angiography for Detection of Coronary Artery In-Stent Restenoses***

Author	Total No. of stents	Stents excluded†	Prevalence (%)	Sensitivity (%)	Specificity (%)
Schuijf (7)	65	15 (23%)	18	78	100
Kitagawa (8)[$]	35	19 (54%)	31	100	100
Cademartiri (9)[¶]	76	2 (3%)	8	83	99
Gaspar (15)[¶]	111	0	20	64	88
Gilard (10)[§]	29	2 (7%)	15	100	100
van Mieghem (11)[§]	74	0	15	91	87
Gilard (13)[¶]	232	0	9	50	53
Rixe (12)[¶]	102	0	12	50	57

* The table (modified from Dewey and Hamm, Röfo 2007) lists only those studies in which the sensitivity and specificity for the detection of in-stent restenoses are given in comparison to conventional coronary angiography and which investigated CT scanners with at least 12 simultaneous detector rows.

† Number of stents excluded from the calculation of accuracy due to poor image quality. This procedure is in contrast to the recommendations for the conduct and publication of diagnostic studies of the STARD initiative (16).

[$] Only a subgroup of the study patients underwent conventional coronary angiography.

[§] The study included only stents of the left main branch. This reflects the great differences that exist between published studies, which is why no accumulation of the accuracy data for the detection of in-stent restenoses over all studies was done.

[¶] These studies also included (a small number of) stents in venous coronary bypass grafts.

Implications for the Design of the Present Study

As shown above the diagnostic performance of MSCT for detection of in-stent restenoses is rather variable and in some studies a remarkable number of patients/stents was excluded from analysis because of poor image quality. This is why the diagnostic performance of MSCT is overestimated. On the other hand, only with 320-slice information in regards to blood flow based on the 4D nature of the scanner are available as myocardial perfusion information (with and without adenosine) and are likely to further improve diagnostic performance. Thus, we expect a diagnostic per-patient accuracy of 87% in our study. Most importantly, a fair analysis of the

clinically relevant performance of MSCT needs to be conducted in an intention-to-diagnose fashion (including all stents regardless of size, location, and image quality). This approach will be used in the present study.

In conclusion, the potential clinical benefit of 320-slice CT for detection of in-stent restenoses should be analyzed using a combined assessment of myocardial perfusion and angiography, in those patients were coronary CT angiography is not evaluable, in comparative study with conventional coronary angiography as the gold standard and an intention-to-diagnose design to represent the clinical value of this new test most appropriately.

4 Trial Objectives

Primary Objective

The primary objective of this study is to analyze the diagnostic accuracy of MSCT using 320 simultaneous detector rows for coronary CT angiography (CTA) and myocardial perfusion (CTP) for the detection of coronary artery in-stent restenoses in comparison to conventional coronary angiography.

The hypothesis will be to demonstrate a per-patient diagnostic accuracy of 320-slice CTA with CTP significantly greater than 75% at an expected per-patient diagnostic accuracy of 87%. For this purpose at a power of 80% and an alpha level of 0.05 altogether 90 patients will have to be recruited.

Secondary Objectives

1. To analyze per-patient sensitivity, specificity, and receiver-operating characteristic curves.
2. To analyze per-stent accuracy, sensitivity and specificity, and and receiver-operating characteristic curves. For this analysis a statistical adjustment for clustering of stents within patients will be conducted using nonparametric analysis of variance.
3. To analyze interobserver variability in detection of coronary artery in-stent restenoses using MSCT.
4. To compare diagnostic accuracy for stents in different locations (coronary vessels), of different size and length, and for stents made of different materials.
5. To analyze the influence of different scan (heart rate, heart rate variability) and patient characteristics (gender, age, diabetes, body mass index) on image quality and diagnostic accuracy of MSCT.
6. To compare procedural complications of MSCT and conventional coronary angiography. These complications will include.
   - death,
   - stroke, and
   - myocardial infarction and
   - moderate to severe groin hematoma, groin pain, infections, allergies, thromboses, and arteriovenous fistula or other complications (if prolonging the in-hospital stay significantly [by at least 24 hours]).

7. To analyze the cost-effectiveness using the primary and secondary efficacy data, the QALY data, and cost data derived from the trial.
8. To analyze patient preference and satisfaction with and comfort during the imaging tests as recently described (Schönenberger et al. PLoS ONE 2007).
9. To compare the amount of contrast agent and radiation exposure and the room time required (excluding time for interventions) of both tests.
10. In addition, noncoronary causes of symptoms (e.g. pulmonary embolism, aortic dissection, pericarditis, pleural effusion, pneumonia, or hiatal hernia) will be recorded to analyze whether the cross-sectional imaging capabilities of MSCT might lead to a higher detection rate of those important incidental findings.
11. To analyze which image quality in MSCT coronary angiography is required to directly reliably triage patients with suspected coronary artery in-stent restenoses.
12. To analyze the correlation and agreement between MSCT coronary angiography and conventional coronary angiography (using quantitative analysis) for estimation of the percent diameter stenosis.

5 Trial Design

Number of Participants and Duration

90 adult men and women age 40 years or older with suspected coronary artery in-stent restenoses scheduled to undergo conventional coronary angiography will be included in this clinical trial and will be analyzed according to the intention-to-treat principle. The study will be conducted at the Charité and the enrollment period is expected to last about 8 to 12 months.

Study Design

This is a prospective, comparative, intention-to-diagnose trial in patients with suspected coronary artery in-stent restenoses based on clinical or findings of other noninvasive tests according to the STARD statement. The current analysis will be performed in the intention-to-diagnose population without excluding patients or stents because of poor image quality. This study consists of three periods: Selection of Patients (see Chapter 6) and Imaging (see Chapter 7). For each patient, it is anticipated that the Selection period will last less than 1 day and will be followed by the Imaging period (less than 1 week).

Withdrawal

Reasons for early withdrawal from a study may include but are not limited to:

1. Patient withdraws consent.
2. Any medical condition or situation that in the opinion of the investigator exposes the patient to significant risk by continuing the study.
3. Any serious adverse event, clinically significant event, or severe laboratory abnormality that may significantly increase the patient's risk in continuing in the study.

6 Selection of Patients

Screening

Prospective patients with suspected coronary artery in-stent restenoses scheduled or planned to undergo further clinical evaluation with coronary angiography (within a maximum of 14 days) will be screened by personnel for possible inclusion into the study. The protocol inclusion and exclusion criteria will be reviewed for each potential participant. If the patient appears to be eligible for the study based on the initial evaluation, and will be undergoing clinically indicated coronary angiography, informed consent will be obtained. Baseline information and history will be collected after informed consent has been obtained using the appropriate CRFs (see Appendix 1). In addition, we will maintain an ongoing log for all patients who were screened for the study and reasons for not being enrolled. Consecutive patients with suspected restenoses will be included to ensure that the population studied is representative of the target population.

Informed Consent

Written informed consent (using the consent form, CF, see CRFs Appendix 1) will be obtained from the patient. The consent process should be in accordance with local institutional standards. In general, the informed consent process involves and explanation of the purpose of the research study, a description of the procedures to be followed during the course of the study, the potential risks/benefits and the expected duration of the patient's participation. The patient should be provided a sufficient opportunity to review the CF and ask questions. Each patient will be assured that he/she is free to withdraw from the study at any time. The original informed consent must be kept on file by the investigator with the patient's record. A copy of the informed CF is to be given to each study patient.

Written Institutional Review Board (IRB) approval for the protocol and the informed CF will be obtained by the coordinating investigator. Any amendments to the protocol, as well as associated CF changes, must be submitted to the IRB, and written approval will be obtained prior to implementation.

Inclusion Criteria

1. Suspected coronary artery in-stent restenoses based on clinical findings or findings on other noninvasive imaging tests and planned coronary angiography within the next 14 days. This clinical indication will be made by cardiologists. The activities of the cardiologists will be overseen and directed by the Cardiology Investigator of the Trial (Professor Karl Stangl). All coronary artery stents (regardless of type and size) will be

eligible for inclusion in the trial. No maximum number of stents per patient will be used to exclude patients.

2. Able to understand and willing to sign the Informed CF.

Exclusion Criteria

1. Creatinine of above 2.0 mg/dl
2. Age below 40 years
3. Women of child bearing potential (no hysterectomy, no menopause, or menopause since less than 12 months) must demonstrate a negative pregnancy test performed within 24 hours before MSCT or conventional coronary angiography.
4. Atrial fibrillation, uncontrolled tachycardia, A-V block II or III degree or other non-sinus rhythms
5. Body weight >300 kg (because of the weight limit of the CT)
6. Coronary artery bypass graft/s
7. Inability to hold the breath for 10 seconds
8. hypotension < 80 mmHg systolic
9. unstable angina pectoris, acute myocardial infarction < 48h
10. continuous therapy with dipyridamol (because of adenosine for CT perfusion)
11. AV-Block II oder III °

Patients with a known allergoid reaction to iodinated contrast agents in the past will not be excluded from the trial if they are considered to have a clinical indication for conventional coronary angiography but will be premedicated according to local standards using 250 mg of Solu-Decortin and 8 mg of Fenistil intravenously. An age limit of 40 years will be used as a balance between increased radiation exposure risks in younger patients and the necessity to include a representative spectrum of patients with suspected in-stent restenoses in the coronaries. Patients with a heart rate above 65 beats per min receive oral beta blockers because with beta blockers the heart rate can be reduced by an average of 10 beats and thus a maximum a heart rate of 60-70 beats per minute is expected during scanning which should give at least an adequate image quality using the most recent reconstruction approaches (17, 18). Patients with atrial fibrillation or uncontrolled tachyarrhythmia will also be excluded, because a regular heart beat is also essential for sufficient image quality with MSCT. Patients must be able to hold their breath for the duration of the MSCT scanning period (10 s). Because of the use of adenosine for perfusion, patients with systolic hypotension (<80 mmHg) and continuous therapy with dipyridamol cannot participate.

Medical History

A medical history and demographic information will be collected on all patients during the Selection and Imaging period. The history will include details regarding all past and current illnesses, past surgical/medical procedures, and concomitant medication usage. Allergies and potential past/recent adverse drug reactions will also be reviewed. Details regarding cardiovascular risk factors and habits, including smoking and alcohol use, nutrition, physical activity, and quality of life will be collected at least during the Imaging Period. Demographic information including age, race, gender, height, and weight will be collected on all patients during this period.

Physical Examination

A targeted physical examination will be performed in the Selection period. The examination will include vital signs: blood pressure and pulse. A targeted physical examination (heart, lungs, and extremities) will be performed at screening. Vital signs will be obtained immediately prior to MSCT scanning and will be repeated just after scanning. Vital signs will be monitored during/following MSCT if beta-blockers are given per local standards. The patient will also be observed for at least 2 days after study-related procedures for any new clinical signs and complications.

Electrocardiography

A standard 12-lead ECG will be performed within 2 days prior to the MSCT. This is reviewed prior to inclusion to ensure no ECG-related exclusion criterion is present. In addition, after the patient has been placed on the ECG in the CT scanner room, the rhythm will again be reviewed prior to imaging.

Baseline Laboratory Tests

Blood will be obtained either at the time of laboratory testing, MSCT (during IV placement) or at the time of cardiac catheterization, for cholesterol profile and serum biomarkers. A locally accepted/certified clinical pathology laboratory will perform the blood and urine tests specified by the protocol within 24 hours before CT. Serum chemistry profile will have to include BUN, creatinine, glucose, sodium, potassium, chloride and bicarbonate. If appropriate, a pregnancy test must also be performed. A qualifying serum creatinine must be performed immediately (maximum of 24 hours) before MSCT.

Further Baseline Tests

Standard two-dimensional transthoracic echocardiography is encouraged as a baseline examination but is not a condition for participation. The results of tests for detection of myocardial ischemia will be recorded using CRFs (see Appendix 1) but will also not be a requirement for participation.

7 MSCT

MSCT coronary angiography is to be performed within 14 days after inclusion of patients for safety reasons. In no instance this time period is allowed to be delayed. Also for safety reasons and reliable comparability of the two tests the time between MSCT and conventional coronary angiography should not be longer than 14 days.

MSCT

All inclusion/exclusion criteria will be reviewed again just prior to the MSCT. Each participant will be clinically evaluated briefly before and following imaging.

Preparation

Patients will be instructed to lay still on the examination table and to hold the breath after instruction (inspiration instruction) for the required period. Valsalva maneuvers should be avoided by breathing in only to a maximum of 80% of the entire capacity. An ECG will be placed on the anterior hemithorax to allow retrospective gating of the images to the ECG tracing.

Nitroglycerine (glycerol trinitrate, 0.8 mg glycerol trinitrate [nitroglycerine], Nitrolingual N Spray, Pohl-Boskamp) shall be given sublingually prior to MSCT coronary angiography as recently described (19) if no contraindications to nitroglycerine are present (severe aortic stenosis, hypertrophic obstructive cardiomyopathy, intake of inhibitors of phosphodiesterase within the last 24 hours, e.g. sildenafil, and severe hypotension, i.e. systolic blood pressure below 100 mm Hg). In patients with contraindications to nitroglycerine MSCT will be conducted without nitroglycerine and the patients will not be excluded from the trial. Patients on chronic therapy with oral or topical long acting nitrates may and should continue their regular use.

Patients were instructed to avoid caffeine and black tea prior to scanning. Patients on beta blockers should continue them during the study. Additional intravenous and/or oral beta blockers (according to the MSCT SOP) may be given prior to the MSCT in order to reduce heart rate to ≤ 65 beats per minute during scanning. Betablocker and nitroglycerine administration will be performed according to the SOP for performance of MSCT (see Appendix 2). Atenolol (50-150 mg) is recommended as the oral beta blocker approximately 1 hour prior to MSCT in patients with a resting heart rate during the physical examination of at least 65 beats per minute. In patients with a resting heart rate of at least 60 immediately prior to CT scanning on the examination table further intravenous beta blockers (recommendation: Esmolol [Brevibloc] at a

maximum dose of 600 mg, maximum of 10-30 mg per minute) can be given.

Patients on metformin or other metformin-containing agents may participate in the study. The patient's clinical physician or site should follow their local standard of care in regards to discontinuation/re-initiation of metformin when intravenous contrast agent administration is planned to be administered. It is generally encouraged that patients with an increased creatinine level (above 1.5 mg/dl) stop their metformin for 2 days following contrast injection (according to the ESUR guideline).

Patients with known allergoid reactions to iodinated contrast agents shall undergo premedication according to local guidelines.

Scanning

CT scanning will be performed on 320-slice scanner with the patient in supine position and holding his or her breath in submaximum inspiratory position. Patients will be positioned off-center to the right to place the heart in the center of the scan field. Electrocardiogram leads will be positioned outside the scan range and arms were placed above the head to improve image quality. The 320-mm scan field-of view (M) of the CT scanner will be used for image acquisition. First, an anteroposterior scanogram (120 kv, 50 mA) will be obtained for further planning. Based on the dimensions of the heart on this scanogram the scan length of the subsequent CT coronary angiography will be defined.

Contrast Agent

MSCT requires contrast agent injection into an antecubital vein (Iomeron, 400 mg/ml, Bracco). An intravenous line will be placed for the administration of the contrast agent. This will be preferably an 18 or 20 gauge intravenous line placed in the right arm. Approximately 50-70 ml of an intravenous contrast will be administered during each of the two CT studies (coronary arteries by CTA and myocardial perfusion by CTP). The flow of the contrast agent will be 5 ml/s with the exception of patients with a body weight of <60 kg – these patients will receive the intravenous contrast agent with a flow rate of 4 ml/s. A region of interest in the aorta will be used to track contrast agent transit time. An automated breathhold command ("breathe in and hold your breath", 4-sec breathhold) will be given when an absolute increase of 200 Hounsfield units in the descending aorta after contrast agent injection had been detected with the automatic sure-start option of the scanner. The contrast agent will be injected in two phases: the first phase will be 50-70 ml of contrast and the second phase will be 40 ml of saline. This will be

administered through an automated power injector following a saline injection (biphasic) in a standardized fashion.

Scanning and Reconstruction

The stationary prospectively triggered scan of the coronary arteries shall cover the range from approximately 1 cm above the left atrium to the base of the heart. In addition, a single axial slice will be acquired at the largest diameter of the heart, which served to define the reconstruction field-of view to be used for all subsequent reconstructions. The size of this field-of view will be in the range of 180 to 220 mm in order to achieve an in-plane pixel size of approximately 0.4*0.4 mm^2 at an image matrix of 512 by 512. The reconstruction field-of view will be set to cover the entire heart and the descending aorta and will be used for CT coronary angiography. All MSCT scans will be performed at 0.5 mm detector collimation. The reconstructed slice thickness will be allowed to be 0.5 mm while the reconstruction interval will be allowed to be between 0.4 and 0.5 mm. The reconstructed FOV (round) shall be set at a maximum of 220 mm and a minimum of 180 mm in size. At the time of the MSCT, the images of the lungs and mediastinum (at large FOV, at least 320 mm) with a maximum reconstructed slice thickness of 5 mm (recommendation: 3-5 mm) will be immediately reviewed at the site for non-cardiac findings according to the MSCT SOP. The Radiology investigators will report these non-cardiac findings to the patient's clinical physician and to the patient in a timely fashion to immediately direct further necessary management.

For CTP imaging patients will receive adenosine at a standard dose (140 µg/kg/min) through an intravenous line in the antecubital fossa of the left arm at least 20 min after nitrogylcerin for CTA. Stress CTP will be performed 4:30 min after initiation of the continuous adenosine infusion.

Following the MSCT study, the patient will be monitored per local standard for at least 2 days to identify procedural complications. At least one post-MSCT set of vital signs will be obtained. The IV line will then be removed after the patient has been felt to have been adequately monitored.

Other Information

If the patient has had an interim change in cardiac clinical status (e.g. worsening of symptoms requiring immediate catheterization without CT), then the patient will be excluded from the analysis.

The MSCT raw data and reconstructed data will be saved at each site for at least 30 years after completion of the study.

Non-cardiac Findings on MSCT

MSCT during the course of this study is being performed for the purpose of coronary artery imaging and assessment of myocardial perfusion. Partially inclusive in these studies will be images of non-coronary structures. Although the CT performed for purpose of this study is performed to optimize coronary and myocardial imaging primarily, other structures will be visualized and should be interpreted. These interpretations should be made with the understanding that they are likely limited by the technique used specifically for the study. The CT images shall be reviewed immediately by a radiologist. Interpretations of the non-cardiac structures should be communicated with the patient, patient's physician, primary care physician, or other pertinent health care provider. Immediate communication is important in the view of potential significantly abnormal or concerning findings. In addition, the findings will be noted on the MSCT CRFs. It is the Radiology investigators who are responsible for the appropriate reading and communications of these non-cardiac CT findings.

8 Assessment of Efficacy

Primary Efficacy Endpoints

The hypothesis of the present trial will be that for combined CTA and CTP the per-patient diagnostic accuracy of 320-slice CT is significantly greater than 75% at an expected per-patient diagnostic accuracy of 87%.

Secondary Efficacy Endpoints

Diagnostic accuracy on other levels (e.g. per-stent) will be conducted. Image quality will be analyzed as well as patient acceptance, cost-effectiveness, non-coronary findings, and radiation dose. Also procedural complications of the two tests will be compared.

9 Assessment of Safety

Adverse Events Monitoring for MSCT

Safety monitoring of the MSCT examination (and if performed MRI) will be performed. This will include monitoring of adverse events possibly related to study-related procedures; such as CT contrast administration, radiation exposure, and medications used for the MSCT (such as beta-blockers and nitroglycerine). Clinical laboratory tests (e.g., creatinine) will be reviewed. Assessment of allergic reactions will be performed.

The safety profile and known side effects and expected adverse events related to contrast media have been well described in the literature. Known and anticipated events include, but are not limited to, allergic reaction (mild or severe), anaphylaxis, pruritus, rash, renal impairment, renal failure, contrast-induced nephropathy, vasovagal reaction. Known risks of intravenous line placement include bleeding, infection, tissue or nerve injury, and vasovagal reaction. Known risks related to beta-blocker medication include, but are not limited to, hypotension, bradyarrhythmia, allergic reaction, bronchospasm, and precipitation of reactive airway disease, heart block. Known risks of nitroglycerine use include headache, reduction in blood pressure, hypotension. These rare but important potential adverse events will be monitored at each site using the corresponding CRFs.

Laboratory Test Results

All laboratory values must be reviewed and appraised by the investigator or research personnel for clinical significance. For any abnormal laboratory value considered to be new since baseline and clinically significant, details must be provided on the Laboratory Adverse Event case report form. This will include whether the event is considered serious, the relationship to the MSCT or MRI contrast agent or other agents, the action taken, and patient outcome. Significant abnormal values occurring during the study are to be followed until repeat values return to normal, stabilize, or are no longer considered clinically significant.

Clinical Adverse Events

All clinical adverse events will be recorded on the Adverse Events (AE) page of the CRF with details including seriousness, date of occurrence, intensity, time of onset/stop, relationship to study (drug, contrast, procedure), action taken (with respect to study-related drug or study-related procedure only), and patient outcome.

Serious Adverse Events

Serious adverse events will be recorded and classified according to the following categories.

1. Fatal;
2. Is life threatening?
3. Results in persistent or significant disability or incapacity
4. Is a congenital anomaly or birth defect?
5. Requires inpatient hospitalization or prolongation of existing hospitalization with the following exceptions:
    a. Preplanned (prior to study), unless hospitalization is prolonged
    b. Ambulatory treatment units or <24 hour re-hospitalizations
    c. Hospitalization for elective procedure
    d. Emergency room visit.

The procedure of reporting of Serious Adverse Events following MSCT is as follows:

1. Report any serious adverse events to the coordinating investigator within 24 hours of knowledge of event.
2. Report any serious adverse event to the IRB according to the local IRB procedures.
3. Complete appropriate Event Form for any complication and/or serious advents.
4. Attach physician /nurse notes or summaries regarding the event of to the Event Form.
5. Report of a patient death must be accompanied by a brief statement of the pertinent details, the death record, death certificates, and autopsy report (if performed).

11 Statistics

Since the benefit of the non-invasive technology, if it exists, should not be withhold from potential beneficiaries for unreasonably long time, and on the other hand the technology might infiltrate medical practice without scientific evidence of benefit, if trial time is too long, it was agreed to aim at a study size allowing for getting results within a 2 years period. Power should be at least 80% with a type I error of $\alpha = 0.05$ (two-sided).

The primary objective of this study is to analyze the diagnostic accuracy of MSCT using 320 simultaneous detector rows for detection of coronary artery in-stent restenoses in comparison to conventional coronary angiography.

We expect a per-patient diagnostic accuracy of combined CTA and CTP of 87% in comparison to conventional coronary angiography. 90 patients are needed to detect these differences with a power of 80% and an alpha level of 0.05.

The statistical design and all statistical analyses will comply with the International Conference on Harmonization (ICH) Guidance for Industry: E9 Statistical Principles for Clinical Trials.

The assumptions for diagnostic accuracy of MSCT are based on the previous studies published (**Table 1**) and the expected improvement using 320-slice CT.

Secondary endpoints will be evaluated using t-tests, Mann-Whitney-U-tests, χ^2-tests, or survival analysis according to scaling. Subgroups (gender, age groups, symptoms) will be analyzed exploratively.

12 Quality Control and Assurance

Methods Against Bias

Diagnostic accuracy trials are significantly influenced by selection and verification bias. To avoid these biases we will include a consecutive cohort of patients which will thus be representative of patients with suspected coronary artery in-stent restenoses. To avoid verification bias MSCT will always be performed prior to conventional coronary angiography. This way it will be avoided that patients with positive findings on invasive imaging and the clinically driven necessity to perform an MSCT will included in the trial. Also all patients will undergo conventional angiography regardless of the result of MSCT coronary angiography.

A primary concern is data quality with regard to transcription of original data from the patients' records to the CRFs. Thus the accuracy of every 10th data set will be compared with the original patient's files.

The coordinating investigator will assure the timely collection of complete and accurate data. There are two major steps associated with ensuring that accurate and complete data will be collected: 1) all persons associated with data collection should be properly trained and familiarized with the tasks that they are to perform; and 2) performance of the required procedures should be monitored and large deviations from study norms investigated.

Charité staff will prepare monthly patient recruitment reports showing the number of patients recruited for that week and cumulatively. In addition, a comparison to recruitment goals will be included. These reports allow the study leadership to monitor the recruitment and follow-up efforts. It is important for the statistical precision of the study to recruit all of the patients suggested by the sample size calculations and to obtain as much follow-up data as possible. In addition to the recruitment reports, a quarterly performance report will include information on the number and percent of forms or data delinquent.

Written CRFs will be used to capture data electronically. The data entry system program provides for editing of data, which includes range checking, table look-up for value accuracy, and intra and inter-form logical consistency checks. If an error occurs, the system automatically notifies the user, and if the error cannot be corrected immediately, errors will be printed out in hard copy for resolution by the study data manager.

Quality assurance procedures will be implemented at every level of this study. A quality assurance procedure will also be implemented in the area of data entry, where certain key fields on each form will be also reviewed a third time by the data manager after processing. A report on quality assurance measures and activities will be prepared every six months for review. This report will include information on protocol violations, missing and delinquent CRF submission.

Original MSCT data as well as conventional coronary angiography films must be maintained and kept for a period of at least 30 years according to the Deutsche Röntgenverordnung (see information in the patient CF).

SOPs

Standard operating procedures (SOPs) will be set up for the following pivotal and decisive aspects and actions during the trial: performance and analysis of MSCT.

13 Ethics

General

The study will be conducted in accordance with the protocol, the current revision of the Declaration of Helsinki, the current guideline for Good Clinical Practice, and with the applicable regulatory requirements. The study protocol will have to be approved by the institutional review boards of each participating center. MSCT exposes the patients to a radiation dose of approximately 15 to 20 mSv. To ensure adherence to legal regulations and safety of the patients, the Federal Department for Radiation Protection will review the study protocol. Iodinated contrast agents will be used for MSCT. These agents have a well-known low risk profile (30). However, these agents will be administered twice for MSCT. Patients will benefit from the detection of noncoronary causes of their symptoms by MSCT.

Due to the importance of protecting study data at the Charité, access to all computer files and programs will be restricted to authorized personnel through the use of passwords and automatic access control at the file level. The Charité intranet has the latest firewall protection and the latest MacAfee virus protection, both of which are updated daily.

Time Intervals between Procedures

MSCT coronary angiography is to be performed within 14 days after inclusion of patients for safety reasons. In no instance this time period is allowed to be delayed. Conventional coronary angiography will be performed within 14 days after MSCT.

Institutional Review Board

The coordinating investigator is responsible for submitting the protocol and Informed Consent Form for the proposed clinical trial to and obtaining approval by the IRB of the Charité. This study is to be conducted according to globally accepted standards of Good Clinical Practice, and in agreement with the latest revision of the Declaration of Helsinki. This protocol and any amendments will be submitted to the properly constituted IRB, in agreement with the local prescriptions, for normal approval of the study conduct.

Federal Department for Radiation Protection

After approval of the study at the IRB of the Charité, the trial protocol and the radiation characteristics of the MSCT scan will be submitted to the Federal Department for Radiation

Protection for review and approval.

Modification of the Protocol

The coordinating investigator must submit necessary protocol modification to the Institutional Review Board and obtain written approval before implementation of any modifications.

14 Publication Policy

The coordinating investigator and the trial statistician are responsible for the writing of the manuscript concerning the main objective of the trial. This shall be done within a period of not more than 12 months after obtaining the final data. At least 1 month prior to submission of the manuscript the manuscript draft will be submitted to the other investigators for review and approval. Authors of all the manuscripts generated from the study will need to fulfill all of the following three criteria according to the ICMJE Uniform Requirements for Manuscripts Submitted to Biomedical Journals:

1. substantial contributions to conception and design, or acquisition of data, or analysis and interpretation of data;
2. drafting the article or revising it critically for important intellectual content; and
3. final approval of the version to be published.

Because of the fact that 3 departments are participating in the trial a maximum of two authors will be allowed per department. In addition, the corresponding author, who directly responsible for the manuscript drafting and the independent blinded readers of CT and conventional coronary angiography can be authors on the manuscript.

There will be no intermediate publications on the main objective of the trial. Each department investigator or co-investigator agrees not to publish or present on the outcomes of the study in the patient he/she has included. Secondary objectives of the trial can be published prior to publication of the main objectives of the study provided that the coordinating investigator approves this.

15 Organization

Coordination of the study will be conducted by the Department of Radiology at Charité with the support of the trial statistician and the Cardiology and Radiology Department investigators. The overall leadership responsibility of the study is under the direction of the coordinating investigator. The institution of the coordinating investigator (Charité, Radiology) will have the role as the sponsor of the study.

16 References

1. **Klever-Deichert G, Hinzpeter B, Hunsche E, Lauterbach KW.** [Costs of coronary heart diseases over the remaining life time in coronary heart disease cases--an analysis of the current status of coronary heart disease cases in Germany from the social perspective]. *Z Kardiol*. 1999;88(12):991-1000.
2. **Tatum JL, Jesse RL, Kontos MC, et al.** Comprehensive strategy for the evaluation and triage of the chest pain patient. *Ann Emerg Med*. 1997;29(1):116-25.
3. **Association AH.** *2002 Heart and Stroke Statistical Update* Dallas, Texas: American Heart Association; 2001.
4. **Dewey M, Hamm B.** Cost-effectiveness of coronary angiography and calcium scoring using CT and stress MRI for diagnosis of coronary artery disease. *Eur Radiol*. 2006:in revision.
5. **van Buuren F, Mannebach H, Horstkotte D.** 20th report of performance data from heart catheterization laboratories in Germany. *Z Kardiol*. 2005;94(3):212-5.
6. **Noto TJ, Jr., Johnson LW, Krone R, et al.** Cardiac catheterization 1990: a report of the Registry of the Society for Cardiac Angiography and Interventions (SCA&I). *Cathet Cardiovasc Diagn*. 1991;24(2):75-83.
7. **Schuijf JD, Bax JJ, Jukema JW, et al.** Feasibility of assessment of coronary stent patency using 16-slice computed tomography. *Am J Cardiol*. 2004;94(4):427-30.
8. **Kitagawa T, Fujii T, Tomohiro Y, et al.** Noninvasive assessment of coronary stents in patients by 16-slice computed tomography. *Int J Cardiol*. 2006;109(2):188-94.
9. **Cademartiri F, Mollet N, Lemos PA, et al.** Usefulness of multislice computed tomographic coronary angiography to assess in-stent restenosis. *Am J Cardiol*. 2005;96(6):799-802.
10. **Gilard M, Cornily JC, Rioufol G, et al.** Noninvasive assessment of left main coronary stent patency with 16-slice computed tomography. *Am J Cardiol*. 2005;95(1):110-2.
11. **Van Mieghem CA, Cademartiri F, Mollet NR, et al.** Multislice spiral computed tomography for the evaluation of stent patency after left main coronary artery stenting: a comparison with conventional coronary angiography and intravascular ultrasound. *Circulation*. 2006;114(7):645-53.
12. **Rixe J, Achenbach S, Ropers D, et al.** Assessment of coronary artery stent restenosis by 64-slice multi-detector computed tomography. *Eur Heart J*. 2006;27(21):2567-72.
13. **Gilard M, Cornily JC, Pennec PY, et al.** Assessment of coronary artery stents by 16 slice computed tomography. *Heart*. 2006;92(1):58-61.
14. **Kondo C, Mori S, Endo M, et al.** Real-time volumetric imaging of human heart without electrocardiographic gating by 256-detector row computed tomography: initial experience. *J Comput Assist Tomogr*. 2005;29(5):694-8.
15. **Gaspar T, Halon DA, Lewis BS, et al.** Diagnosis of coronary in-stent restenosis with multidetector row spiral computed tomography. *J Am Coll Cardiol*. 2005;46(8):1573-9.
16. **Bossuyt PM, Reitsma JB, Bruns DE, et al.** Towards complete and accurate reporting of studies of diagnostic accuracy: The STARD Initiative. *Ann Intern Med*. 2003;138(1):40-4.
17. **Hoffmann MH, Shi H, Manzke R, et al.** Noninvasive coronary angiography with 16-detector row CT: effect of heart rate. *Radiology*. 2005;234(1):86-97.
18. **Dewey M, Laule M, Krug L, et al.** Multisegment and halfscan reconstruction of 16-slice computed tomography for detection of coronary artery stenoses. *Invest Radiol*. 2004;39(4):223-9.
19. **Dewey M, Hoffmann H, Hamm B.** Multislice CT Coronary Angiography: Effect of Sublingual Nitroglycerine on the Diameter of Coronary Arteries. *Fortschr Röntgenstr*.

2006;178(6):in press.
20. **Kim RJ, Wu E, Rafael A, et al.** The use of contrast-enhanced magnetic resonance imaging to identify reversible myocardial dysfunction. *N Engl J Med*. 2000;343(20):1445-53.
21. **Cerqueira MD, Weissman NJ, Dilsizian V, et al.** Standardized myocardial segmentation and nomenclature for tomographic imaging of the heart: a statement for healthcare professionals from the Cardiac Imaging Committee of the Council on Clinical Cardiology of the American Heart Association. *Circulation*. 2002;105(4):539-42.
22. **Miller S, Simonetti OP, Carr J, Kramer U, Finn JP.** MR Imaging of the heart with cine true fast imaging with steady-state precession: influence of spatial and temporal resolutions on left ventricular functional parameters. *Radiology*. 2002;223(1):263-9.
23. **Griswold MA, Jakob PM, Heidemann RM, et al.** Generalized autocalibrating partially parallel acquisitions (GRAPPA). *Magn Reson Med*. 2002;47(6):1202-10.
24. **Lorenz CH, Walker ES, Morgan VL, Klein SS, Graham TP, Jr.** Normal human right and left ventricular mass, systolic function, and gender differences by cine magnetic resonance imaging. *J Cardiovasc Magn Reson*. 1999;1(1):7-21.
25. **Yamaoka O, Yabe T, Okada M, et al.** Evaluation of left ventricular mass: comparison of ultrafast computed tomography, magnetic resonance imaging, and contrast left ventriculography. *Am Heart J*. 1993;126(6):1372-9.
26. **Juergens KU, Grude M, Maintz D, et al.** Multi-detector row CT of left ventricular function with dedicated analysis software versus MR imaging: initial experience. *Radiology*. 2004;230(2):403-10.
27. **Dirksen MS, Bax JJ, de Roos A, et al.** Usefulness of dynamic multislice computed tomography of left ventricular function in unstable angina pectoris and comparison with echocardiography. *Am J Cardiol*. 2002;90(10):1157-60.
28. **Simonetti OP, Kim RJ, Fieno DS, et al.** An improved MR imaging technique for the visualization of myocardial infarction. *Radiology*. 2001;218(1):215-23.
29. **Gupta A, Lee VS, Chung YC, Babb JS, Simonetti OP.** Myocardial infarction: optimization of inversion times at delayed contrast-enhanced MR imaging. *Radiology*. 2004;233(3):921-6.
30. **Katayama H, Yamaguchi K, Kozuka T, Takashima T, Seez P, Matsuura K.** Adverse reactions to ionic and nonionic contrast media. A report from the Japanese Committee on the Safety of Contrast Media. *Radiology*. 1990;175(3):621-8.
31. **Luepker RV, Apple FS, Christenson RH, et al.** Case definitions for acute coronary heart disease in epidemiology and clinical research studies: a statement from the AHA Council on Epidemiology and Prevention; AHA Statistics Committee; World Heart Federation Council on Epidemiology and Prevention; the European Society of Cardiology Working Group on Epidemiology and Prevention; Centers for Disease Control and Prevention; and the National Heart, Lung, and Blood Institute. *Circulation*. 2003;108(20):2543-9.
