# Supplementary material for: The Impact of Different Levels of Adaptive Iterative Dose Reduction 3D on Image Quality of 320-Row Coronary CT Angiography: A Clinical Trial
Source: PLoS One. 2015 May 6;10(5):e0125943. doi: 10.1371/journal.pone.0125943 (PMC4422621; doi:10.1371/journal.pone.0125943)
Supplement: S2 Table — (DOCX) [file pone.0125943.s004.docx]

| **Table S2:** Analysis of the signal | | | | | | | | | | | | | | | | |
| --- | --- | --- | --- | --- | --- | --- | --- | --- | --- | --- | --- | --- | --- | --- | --- | --- |
|  |  |  |  |  |  |  |  |  |  | **ANOVA** | **t-test** |  |  |  |  |  |
|  |  | **FBP/QDS** |  | **MILD** |  | **STD** |  | **STR** |  | **p** | **p1** | **p2** | **p3** | **p4** | **p5** | **p6** |
|  | mean | 155.3 | (53.7) | 155.0 | (53.5) | 152.5 | (53.4) | 149.9 | (53.3) | <0.001 | 1.000 | <0.001 | <0.001 | <0.001 | <0.001 | <0.001 |
| **vessel** | Ao | 500.9 | (75.8) | 500.4 | (75.7) | 499.9 | (75.8) | 499.6 | (75.8) | 0.009 | 1.000 | 0.208 | 0.037 | 0.016 | 0.004 | 0.028 |
|  | RCA 1 | 435.0 | (77.7) | 433.0 | (77.4) | 428.3 | (76.6) | 422.7 | (75.3) | <0.001 | 0.181 | <0.001 | <0.001 | <0.001 | <0.001 | <0.001 |
|  | RCA 2 | 384.1 | (78.3) | 383.3 | (80.2) | 378.7 | (79.7) | 374.1 | (79.6) | <0.001 | 1.000 | <0.001 | <0.001 | <0.001 | <0.001 | <0.001 |
|  | RCA 3 | 329.1 | (84.2) | 328.4 | (83.0) | 322.1 | (82.2) | 314.7 | (81.9) | <0.001 | 1.000 | <0.001 | <0.001 | <0.001 | <0.001 | <0.001 |
|  | LM | 460.2 | (71.2) | 459.9 | (71.6) | 456.3 | (70.7) | 452.0 | (69.7) | <0.001 | 1.000 | <0.001 | <0.001 | <0.001 | <0.001 | <0.001 |
|  | LAD 1 | 396.2 | (70.8) | 394.9 | (70.4) | 388.4 | (72.0) | 381.4 | (71.5) | <0.001 | 1.000 | <0.001 | <0.001 | <0.001 | <0.001 | <0.001 |
|  | LAD 2 | 333.9 | (102.7) | 335.3 | (101.7) | 329.1 | (101.9) | 322.7 | (101.5) | <0.001 | 1.000 | 0.020 | <0.001 | <0.001 | <0.001 | <0.001 |
|  | LAD 3 | 251.2 | (89.1) | 253.2 | (85.4) | 246.6 | (86.1) | 241.0 | (86.5) | <0.001 | 0.502 | 0.008 | <0.001 | <0.001 | <0.001 | <0.001 |
|  | LCX 1 | 382.8 | (93.5) | 381.8 | (92.1) | 375.3 | (92.9) | 368.6 | (94.3) | <0.001 | 1.000 | <0.001 | <0.001 | <0.001 | <0.001 | <0.001 |
|  | LCX 2 | 300.6 | (92.1) | 300.8 | (92.3) | 292.5 | (93.9) | 283.1 | (95.5) | <0.001 | 1.000 | 0.005 | <0.001 | <0.001 | <0.001 | <0.001 |
|  | LCX 3 | 218.5 | (84.0) | 221.0 | (85.7) | 213.4 | (86.1) | 205.2 | (84.8) | <0.001 | 0.991 | 0.022 | <0.001 | <0.001 | <0.001 | <0.001 |
| **surr. tissue** | RCA 1 | -70.0 | (16.0) | -70.0 | (17.5) | -68.4 | (16.3) | -66.4 | (15.7) | <0.001 | 1.000 | 0.068 | <0.001 | 0.016 | <0.001 | <0.001 |
|  | RCA 2 | -63.4 | (17.5) | -65.0 | (18.2) | -63.9 | (17.8) | -63.1 | (17.9) | 0.001 | 0.033 | 1.000 | 1.000 | 0.033 | 0.011 | 0.162 |
|  | RCA 3 | -74.9 | (23.8) | -75.4 | (24.2) | -74.5 | (23.0) | -73.5 | (22.2) | 0.009 | 1.000 | 1.000 | 0.076 | 0.066 | 0.019 | 0.051 |
|  | LM | -69.2 | (18.2) | -70.6 | (17.1) | -69.4 | (18.0) | -68.1 | (18.5) | 0.001 | 0.233 | 1.000 | 0.014 | 0.014 | 0.001 | 0.001 |
|  | LAD 1 | -69.3 | (27.4) | -69.2 | (26.6) | -68.1 | (26.4) | -67.1 | (26.5) | 0.002 | 1.000 | 0.305 | 0.011 | 0.041 | 0.006 | 0.007 |
|  | LAD 2 | -85.2 | (17.0) | -85.9 | (17.4) | -85.6 | (16.3) | -84.8 | (15.7) | 0.181 |  |  |  |  |  |  |
|  | LAD 3 | -81.8 | (23.4) | -82.5 | (22.7) | -81.7 | (22.0) | -81.0 | (21.7) | 0.211 |  |  |  |  |  |  |
|  | LCX 1 | -71.6 | (24.6) | -72.2 | (24.6) | -71.5 | (24.1) | -70.9 | (23.8) | 0.135 |  |  |  |  |  |  |
|  | LCX 2 | -79.1 | (21.4) | -79.4 | (21.3) | -78.5 | (21.3) | -77.6 | (21.5) | 0.023 | 1.000 | 1.000 | 0.061 | 0.117 | 0.010 | 0.001 |
|  | LCX 3 | -67.5 | (18.8) | -67.5 | (19.0) | -66.5 | (19.3) | -65.6 | (19.5) | 0.049 | 1.000 | 1.000 | 0.217 | 0.007 | 0.001 | 0.018 |

Values are given in arithmetic mean (SD); reconstruction with filtered back projection/ quantum denoising filtering system (**FBP/QDS)**, adaptive iterative dose reduction (AIDR) 3D mild (**MILD**), standard (**STD**) and strong (**STR**); measurements in the **vessel** and the surrounding tissue (**surr. tissue**) of the proximal (Ao, RCA1, LM, LAD1, LCX1), mid (RCA2, LAD2, LCX2) and distal (RCA3, LAD3, LCX3) coronary segments; First, Repeated Measures ANOVA overall analysis including every measurement point as dependent variable showed p<0.001 (mean p ANOVA). ANOVA for each measurement point, but summarising the 4 reconstructions was done. Only if ANOVA for the separate measurement points showed p≤0.05, t-test was used with a significance level of p=0.002 adapted to the 21 measurement points. Bonferroni correction was automatically performed for the multiple testing with 6 possibilities: **p1** (FBP/QDS-AIDR 3D mild), **p2** (FBP/QDS-AIDR 3D standard), **p3** (FBP/QDS-AIDR 3D strong), **p4** (AIDR 3D mild-AIDR 3D standard), **p5** (AIDR 3D mild-AIDR 3D strong), **p6** (AIDR 3D standard-AIDR 3D strong); **signal** presents the density in Hounsfield Units
