# Supplementary material for: The Impact of Different Levels of Adaptive Iterative Dose Reduction 3D on Image Quality of 320-Row Coronary CT Angiography: A Clinical Trial
Source: PLoS One. 2015 May 6;10(5):e0125943. doi: 10.1371/journal.pone.0125943 (PMC4422621; doi:10.1371/journal.pone.0125943)
Supplement: S3 Table — (DOCX) [file pone.0125943.s005.docx]

| **Table S3**: Analysis of the noise | | | | | | | | | | | | | | | | |
| --- | --- | --- | --- | --- | --- | --- | --- | --- | --- | --- | --- | --- | --- | --- | --- | --- |
|  |  |  |  |  |  |  |  |  |  | **ANOVA** | **t-test** |  |  |  |  |  |
|  |  | **FBP/QDS** |  | **MILD** |  | **STD** |  | **STR** |  | **p** | **p1** | **p2** | **p3** | **p4** | **p5** | **p6** |
|  | Mean | 32.8 | (9.3) | 34.5 | (8.9) | 29.8 | (8.3) | 27.0 | (7.8) | <0.001 | <0.001 | <0.001 | <0.001 | <0.001 | <0.001 | <0.001 |
| **vessel** | Ao | 36.2 | (9.2) | 38.4 | (6.3) | 32.1 | (6.0) | 27.8 | (5.5) | <0.001 | 0.014 | <0.001 | <0.001 | <0.001 | <0.001 | <0.001 |
|  | RCA 1 | 38.0 | (8.3) | 38.4 | (8.5) | 34.4 | (7.5) | 32.2 | (7.1) | <0.001 | 1.000 | <0.001 | <0.001 | <0.001 | <0.001 | <0.001 |
|  | RCA 2 | 34.5 | (8.3) | 35.8 | (8.5) | 32.4 | (8.5) | 30.5 | (8.5) | <0.001 | 0.118 | <0.001 | <0.001 | <0.001 | <0.001 | <0.001 |
|  | RCA 3 | 35.5 | (10.9) | 36.4 | (11.3) | 33.0 | (11.9) | 31.1 | (11.7) | <0.001 | 1.000 | 0.018 | <0.001 | <0.001 | <0.001 | <0.001 |
|  | LM | 38.5 | (10.8) | 38.8 | (10.0) | 34.4 | (9.8) | 31.8 | (9.3) | <0.001 | 1.000 | <0.001 | <0.001 | <0.001 | <0.001 | <0.001 |
|  | LAD 1 | 38.2 | (12.9) | 39.8 | (12.1) | 36.2 | (11.5) | 34.7 | (11.8) | <0.001 | 0.063 | 0.060 | 0.018 | <0.001 | <0.001 | 0.045 |
|  | LAD 2 | 34.6 | (10.4) | 36.2 | (11.3) | 32.7 | (10.4) | 30.7 | (9.6) | <0.001 | 0.119 | 0.025 | <0.001 | <0.001 | <0.001 | <0.001 |
|  | LAD 3 | 32.1 | (10.5) | 33.8 | (11.8) | 30.8 | (11.1) | 29.0 | (10.7) | <0.001 | 0.356 | 0.561 | 0.001 | <0.001 | <0.001 | <0.001 |
|  | LCX 1 | 37.3 | (9.1) | 37.3 | (9.0) | 33.7 | (9.2) | 31.6 | (9.0) | <0.001 | 1.000 | <0.001 | <0.001 | <0.001 | <0.001 | 0.001 |
|  | LCX 2 | 38.3 | (12.3) | 38.8 | (12.3) | 34.6 | (11.1) | 32.1 | (9.8) | <0.001 | 1.000 | 0.001 | <0.001 | <0.001 | <0.001 | <0.001 |
|  | LCX 3 | 34.9 | (8.5) | 36.0 | (8.3) | 31.6 | (7.3) | 28.8 | (6.7) | <0.001 | 0.224 | <0.001 | <0.001 | <0.001 | <0.001 | <0.001 |
| **surr. tissue** | RCA 1 | 31.7 | (8.4) | 35.0 | (8.6) | 28.3 | (7.8) | 24.4 | (7.6) | <0.001 | <0.001 | <0.001 | <0.001 | <0.001 | <0.001 | <0.001 |
|  | RCA 2 | 27.5 | (8.2) | 30.6 | (7.8) | 25.6 | (6.8) | 22.4 | (5.8) | <0.001 | <0.001 | 0.004 | <0.001 | <0.001 | <0.001 | <0.001 |
|  | RCA 3 | 29.3 | (10.0) | 31.4 | (8.2) | 26.2 | (7.9) | 22.9 | (7.3) | <0.001 | 0.026 | <0.001 | <0.001 | <0.001 | <0.001 | <0.001 |
|  | LM | 28.4 | (6.8) | 31.4 | (6.2) | 25.6 | (5.9) | 22.0 | (5.5) | <0.001 | 0.001 | 0.001 | <0.001 | <0.001 | <0.001 | <0.001 |
|  | LAD 1 | 29.2 | (7.3) | 31.4 | (7.1) | 25.7 | (6.1) | 22.0 | (5.3) | <0.001 | 0.050 | <0.001 | <0.001 | <0.001 | <0.001 | <0.001 |
|  | LAD 2 | 25.7 | (7.3) | 28.3 | (7.0) | 23.1 | (5.9) | 20.3 | (5.1) | <0.001 | 0.001 | 0.001 | <0.001 | <0.001 | <0.001 | <0.001 |
|  | LAD 3 | 24.4 | (7.6) | 26.0 | (6.8) | 21.6 | (6.0) | 19.4 | (5.3) | <0.001 | 0.073 | 0.001 | <0.001 | <0.001 | <0.001 | <0.001 |
|  | LCX 1 | 30.4 | (8.4) | 31.8 | (7.6) | 26.2 | (6.6) | 22.8 | (6.2) | <0.001 | 0.291 | <0.001 | <0.001 | <0.001 | <0.001 | <0.001 |
|  | LCX 2 | 32.1 | (8.9) | 33.8 | (8.2) | 28.4 | (7.4) | 24.8 | (6.8) | <0.001 | 0.110 | <0.001 | <0.001 | <0.001 | <0.001 | <0.001 |
|  | LCX 3 | 32.8 | (11.4) | 35.5 | (9.9) | 29.5 | (9.9) | 25.3 | (9.5) | <0.001 | 0.002 | <0.001 | <0.001 | <0.001 | <0.001 | <0.001 |

Values are given in arithmetic mean (SD); reconstruction with filtered back projection/ quantum denoising filtering system (**FBP/QDS)**, AIDR 3D mild (**MILD**), standard (**STD**) and strong (**STR**); measurements in the **vessel** and the surrounding tissue (**surr. tissue**) of the proximal (Ao, RCA1, LM, LAD1, LCX1), mid (RCA2, LAD2, LCX2) and distal (RCA3, LAD3, LCX3) coronary segments; First, Repeated Measures ANOVA overall analysis including every measurement point as dependent variable showed p<0.001 (mean p ANOVA). ANOVA for each measurement point, but summarising the 4 reconstructions was done. ANOVA for the separate measurement points showed p<0.001, so t-test was performed at each measurement point with a significance level of p=0.002 adapted to the 21 measurement points. Bonferroni correction was automatically performed for the multiple testing with 6 possibilities: **p1** (FBP/QDS-AIDR 3D mild), **p2** (FBP/QDS-AIDR 3D standard), **p3** (FBP/QDS-AIDR 3D strong), **p4** (AIDR 3D mild-AIDR 3D standard), **p5** (AIDR 3D mild-AIDR 3D strong), **p6** (AIDR 3D standard-AIDR 3D strong); **noise** is SD of the signal
