# Supplementary material for: The Impact of Different Levels of Adaptive Iterative Dose Reduction 3D on Image Quality of 320-Row Coronary CT Angiography: A Clinical Trial
Source: PLoS One. 2015 May 6;10(5):e0125943. doi: 10.1371/journal.pone.0125943 (PMC4422621; doi:10.1371/journal.pone.0125943)
Supplement: S4 Table — (DOCX) [file pone.0125943.s006.docx]

| **Table S4:** Analysis of the signal-noise-ratio (SNR) and the contrast-noise-ratio (CNR) | | | | | | | | | | | | |
| --- | --- | --- | --- | --- | --- | --- | --- | --- | --- | --- | --- | --- |
|  |  |  |  |  |  |  |  |  |  | **ANOVA** | **t-test** |  |
|  |  | **FBP/QDS** |  | **MILD** |  | **STD** |  | **STR** |  | **p** | **FBP-MILD** | **FBP-STD** |
| **SNR** | Mean | 10.4 | (3.0) | 9.4 | (3.0) | 11.1 | (3.7) | 12.6 | (4.4) | <0.001 | 0.001 | 0.001 |
|  | RCA1 | 12.9 | (4.1) | 11.6 | (2.9) | 13.8 | (3.5) | 15.8 | (4.2) | <0.001 | 0.001 | 0.001 |
|  | RCA2 | 11.5 | (4.4) | 10.4 | (3.2) | 12.4 | (3.9) | 14.1 | (4.7) | <0.001 | 0.001 | 0.001 |
|  | RCA3 | 9.8 | (3.7) | 8.8 | (2.9) | 10.5 | (3.5) | 11.9 | (4.2) | <0.001 | 0.001 | 0.002 |
|  | LM | 13.6 | (4.2) | 12.3 | (2.9) | 14.7 | (3.6) | 16.9 | (4.4) | <0.001 | 0.001 | <0.001 |
|  | LAD1 | 11.8 | (4.0) | 10.6 | (2.9) | 12.6 | (3.6) | 14.3 | (4.2) | <0.001 | 0.001 | 0.001 |
|  | LAD2 | 10.0 | (4.7) | 9.1 | (3.6) | 10.7 | (4.4) | 12.2 | (5.3) | <0.001 | 0.003 | 0.003 |
|  | LAD3 | 7.6 | (4.1) | 6.9 | (3.1) | 8.2 | (3.9) | 9.3 | (4.6) | <0.001 | 0.016 | 0.007 |
|  | LCX1 | 11.2 | (3.9) | 10.1 | (2.8) | 12.0 | (3.5) | 13.7 | (4.3) | <0.001 | 0.002 | 0.002 |
|  | LCX2 | 8.9 | (3.9) | 8.0 | (3.0) | 9.4 | (3.7) | 10.5 | (4.4) | <0.001 | 0.004 | 0.011 |
|  | LCX3 | 6.4 | (3.2) | 5.9 | (2.7) | 6.8 | (3.3) | 7.7 | (3.9) | <0.001 | 0.010 | 0.005 |
| **CNR** | Mean | 12.5 | (4.3) | 11.4 | (3.1) | 13.5 | (3.9) | 15.3 | (4.7) | <0.001 | 0.002 | <0.001 |
|  | RCA1 | 14.9 | (4.4) | 13.5 | (2.9) | 16.0 | (3.6) | 18.2 | (4.3) | <0.001 | 0.001 | 0.001 |
|  | RCA2 | 13.3 | (4.7) | 12.1 | (3.4) | 14.4 | (4.2) | 16.5 | (5.0) | <0.001 | 0.001 | 0.001 |
|  | RCA3 | 11.9 | (4.1) | 10.8 | (2.9) | 12.8 | (3.7) | 14.5 | (4.4) | <0.001 | 0.001 | 0.001 |
|  | LM | 15.6 | (4.6) | 14.2 | (3.1) | 17.0 | (4.0) | 19.4 | (4.9) | <0.001 | 0.002 | <0.001 |
|  | LAD1 | 13.8 | (4.6) | 12.5 | (3.2) | 14.8 | (4.0) | 16.8 | (4.7) | <0.001 | 0.001 | 0.001 |
|  | LAD2 | 12.6 | (5.3) | 11.4 | (3.9) | 13.5 | (4.8) | 15.4 | (5.8) | <0.001 | 0.004 | 0.001 |
|  | LAD3 | 10.0 | (4.3) | 9.1 | (3.2) | 10.8 | (4.0) | 12.2 | (4.8) | <0.001 | 0.009 | 0.002 |
|  | LCX1 | 13.2 | (4.0) | 12.1 | (2.9) | 14.3 | (3.6) | 16.3 | (4.4) | <0.001 | 0.001 | 0.001 |
|  | LCX2 | 11.2 | (4.2) | 10.2 | (3.2) | 11.9 | (3.8) | 13.4 | (4.6) | <0.001 | 0.003 | 0.004 |
|  | LCX3 | 8.4 | (3.3) | 7.7 | (2.7) | 9.0 | (3.2) | 10.1 | (3.9) | <0.001 | 0.006 | 0.001 |

Values are given in arithmetic mean (SD); reconstruction with filtered back projection/ quantum denoising filtering system (**FBP/QDS)**, AIDR 3D mild (**MILD**), standard (**STD**) and strong (**STR**); Repeated Measures ANOVA overall analysis including every measurement point as dependent variable showed p<0.001 (SNR and CNR mean p ANOVA); Because ANOVA for each measurement point, but summarising the 4 reconstructions showed p<0.001, t-test was performed at each measurement point with a significance level of p≤0.005 adapted to the 10 measurement points for both SNR and CNR; The comparisons FBP-STR, MILD-STD, MILD-STR and STD-STR are not integrated in the table because at each measurement point p-value was <0.001 for both SNR and CNR; Bonferroni correction was automatically performed for the multiple testing with 6 possibilities; **SNR**= signal in the vessel measurement point/noise in the aorta ascendens measurement point; **CNR**= (signal in the surrounding tissue of the vessel measurement point - signal in the vessel measurement point)/noise in the aorta ascendens measurement point
