# Supplementary material for: The Impact of Different Levels of Adaptive Iterative Dose Reduction 3D on Image Quality of 320-Row Coronary CT Angiography: A Clinical Trial
Source: PLoS One. 2015 May 6;10(5):e0125943. doi: 10.1371/journal.pone.0125943 (PMC4422621; doi:10.1371/journal.pone.0125943)
Supplement: S5 Table — (DOCX) [file pone.0125943.s007.docx]

| **Table S5:** Analysis of the contour sharpness | | | | | | | | | | | | | | | | |
| --- | --- | --- | --- | --- | --- | --- | --- | --- | --- | --- | --- | --- | --- | --- | --- | --- |
|  |  |  |  |  |  |  |  |  |  | **ANOVA** | **t-test** |  |  |  |  |  |
|  |  | **FBP/QDS** |  | **MILD** |  | **STD** |  | **STR** |  | **p** | **p1** | **p2** | **p3** | **p4** | **p5** | **p6** |
| **CS in pixel** | mean | 8.5 | (2.1) | 8.6 | (2.1) | 8.5 | (1.9) | 8.6 | (1.7) | 0.082 |  |  |  |  |  |  |
|  | RCA | 8.9 | (2.4) | 9.1 | (2.5) | 9.0 | (2.3) | 9.0 | (2.0) | 0.519 |  |  |  |  |  |  |
|  | LMA | 9.9 | (2.1) | 10.1 | (2.0) | 9.9 | (1.9) | 9.9 | (1.7) | 0.187 |  |  |  |  |  |  |
|  | LAD | 7.5 | (1.5) | 7.5 | (1.4) | 7.5 | (1.3) | 7.6 | (1.3) | 0.601 |  |  |  |  |  |  |
|  | LCX | 7.6 | (2.5) | 7.8 | (2.4) | 7.6 | (2.2) | 7.8 | (1.8) | 0.417 |  |  |  |  |  |  |
| **CS in %** | mean | 8.1 | (1.6) | 7.8 | (1.4) | 7.8 | (1.4) | 7.5 | (1.5) | 0.000 | 0.055 | 0.002 | 0.001 | 0.033 | 0.056 | 0.173 |
|  | RCA | 8.0 | (1.8) | 7.7 | (1.4) | 7.6 | (1.4) | 7.2 | (1.2) | 0.001 | 1.000 | 0.907 | 0.013 | 0.728 | 0.014 | 0.008 |
|  | LMA | 6.8 | (1.2) | 6.7 | (1.0) | 6.6 | (1.0) | 6.3 | (0.9) | 0.463 |  |  |  |  |  |  |
|  | LAD | 8.5 | (1.4) | 8.3 | (1.4) | 8.2 | (1.3) | 8.5 | (2.6) | 0.002 | 0.947 | 0.398 | 0.016 | 1.000 | 0.011 | 0.001 |
|  | LCX | 8.8 | (2.0) | 8.7 | (1.9) | 8.6 | (1.8) | 8.1 | (1.3) | 0.002 | 0.483 | 0.158 | 0.004 | 1.000 | <0.001 | 0.001 |

Values are given in arithmetic mean (SD). Evaluation of contour sharpness is based on the difference between 25% and 75% of the maximal gray value (**CS in pixel;** 1mm = 8.292 pixel) and the maximal slope of gray values in the contour (**CS in %)** between filtered back projection/ quantum denoising filtering system (**FBP/QDS)**, AIDR 3D mild (**MILD**), AIDR 3D standard (**STD**) and AIDR 3D strong (**STR**); Repeated Measures ANOVA overall analysis including every measurement point as dependent variable was performed (mean). If p-value was ≤0.05, ANOVA for each measurement point, but summarising the 4 reconstructions was done. Only if ANOVA for the separate measurement points showed p≤0.05, t-test was used with a significance level of p≤0.01 adapted to the 4 measurement points. Bonferroni correction was automatically performed for the multiple testing with 6 possibilities: **p1** (FBP/QDS-AIDR 3D mild), **p2** (FBP/QDS-AIDR 3D standard), **p3** (FBP/QDS-AIDR 3D strong), **p4** (AIDR 3D mild-AIDR 3D standard), **p5** (AIDR 3D mild-AIDR 3D strong), **p6** (AIDR 3D standard-AIDR 3D strong
